# Supplementary material for: Genetic determinants of zinc homeostasis and its role in cardiometabolic diseases
Source: PLoS Genet. 2025 Dec 1;21(12):e1011928. doi: 10.1371/journal.pgen.1011928 (PMC12677789; doi:10.1371/journal.pgen.1011928)
Supplement: S1 File — (PDF) [file pgen.1011928.s001.pdf]

Table of contents

Table of contents **1**
Supplementary Notes **3**
Note A. Genotyping and data pre-processing 3
SKIPOGH 3
CoLaus 3
GCKD 4
Note B. Zinc measurements 4
Note C. Relationship with ABO blood group 6
Note D. Protocol and analyses for animal experiments 8
Dietary zinc supplementation 8
Immunofluorescence analysis 9
RNA extraction and RT-qPCR 10
RNA-sequencing and bioinformatic analysis 11
Supplementary Figures **12**
Figure A. Locus plots of the independent top hits 13
Figure B. Locus plots of the independent top hits 14
Figure C. Kernel-Density plots showing the distribution plasma concentration and urinary zinc excretion across rs3008217 genotypes in SKIPOGH 15
Figure D. Fine-mapping results of region 1 16
Figure E. Fine-mapping results of region 2 17
Figure F. Fine-mapping results of region 3 18
Figure G. Fine-mapping results of region 4 19
Figure H. Fine-mapping results of region 5 20
Figure I. Fine-mapping results of region 6 21
Figure J. Fine-mapping results of region 7 22
Figure K. Fine-mapping results of region 8 23
Figure L. Fine-mapping results of region 9 24
Figure M. Scatterplots of the exposure and outcome SNP associations of the instrumental variables (IVs) used in the forward Mendelian randomization analyses of zinc levels on traits 25
Figure N. Scatterplots of the exposure and outcome SNP associations of the instrumental variables (IVs) used in the forward Mendelian randomization analyses of zinc levels on traits 26
Figure O. Scatterplots of the exposure and outcome SNP associations of the instrumental variables (IVs) used in the forward Mendelian randomization analyses of zinc levels on traits 27

Figure P. RNA-seq analysis of Zn2+ deficient (Def) mouse kidneys compared to control (Ctr)....28
Figure Q. Gene expression levels in human mammary epithelium....29
Figure R. scRNA-seq data in human kidney and breast....30
Supplementary Tables....32
Table A: Urinary zinc excretion and blood zinc excretion genetic effects of the variants with genome-wide significance in either the urine or blood GWAS....32
Table B. GWAS summary statistics used in the bidirectional Mendelian randomization analyses....33
Table C. Mendelian randomization sensitivity analyses using the simple median and simple mode methods of forward causal effects of urinary zinc excretion levels on traits... 34
Table D. Sensitivity analyses of forward MR results of zinc on clinical phenotypes by leaving pleiotropic instrumental variables out....36
Table E. Mendelian randomization sensitivity analyses using the simple median and simple mode methods of reverse causal effects of traits on urinary zinc excretion levels.... 37
Table F. Pearson correlations between allele frequencies (frequency of urinary zinc-increasing allele) and zinc deficiency prevalences....39
Table G. Extract from the GWAS atlas PheWAS database for rs3008217....40
INSPIRE Consortium - Author information....41
References....41

Supplementary Notes

Note A. Genotyping and data pre-processing

SKIPOGH

Genome-wide SNP data was obtained from white blood cells' DNA and generated using the Illumina Human Omni 2.5 platform [1]. We subsequently performed SNP data pre-processing and quality control checks by applying an in-house built algorithm [1–5]. Briefly, after an automatic clustering in GenomeStudio (Illumina Inc. San Diego), we selected samples (participants) with a call rate (proportion of non-missing SNPs) >0.99 to update the SNP statistics. Once re-clustered, we retained markers with a call rate >0.95. The quality control and pre-processing procedures yielded 979 samples with 1,637,659 SNPs, whose call rate was >95%, whose minor allele frequency (MAF) was >2%, and whose Hardy-Weinberg Equilibrium P value was >0.001. We then performed multiple imputation for SNP missing values using the Minimac3 imputation algorithm using the TOPMed r3 imputation panel, yielding 422594786 SNPs of which 8279879 SNPs are available for analyses after allele frequency threshold filtering [6–8].

CoLaus

Genomic data from CoLaus participants was genotyped with the Affymetrix Axiom (GSKBB2) and imputed with the TOPMed reference panel. SNPs & subjects with <97% call rate, MAF<0.1% or deviating from Hardy-Weinberg equilibrium (P<1e-6) were removed.

GCKD

Genomic DNA from GCKD participants was genotyped at 2,612,357 variants using . The haplotype phasing was performed using Eagle 2.4, followed by imputation using Minimac4 and the TOPMed reference panel. On the variant level, SNPs with <96% call rate, imputation quality of r2 ≤ 0.3, MAF < 1% or deviating from Hardy–Weinberg equilibrium (P < 1 × 10−10) and all multi-allelic SNPs were removed [9]. Approximately 7.9 million variants with with MAF > 0.01 were included in GWAS.

Note B. Zinc measurements

Analysis of urine samples from SKIPOGH, CoLaus and GCKD, plasma samples from SKIPOGH cohort and breast milk samples from INSPIRE cohort were performed on a certified method on an Agilent 7800 instruments equipped with quadrupole detector (Agilent Technologies, Santa Clara, CA, USA). Measured isotopes were 7Li, 9Be, 27Al, 51V, 53Cr, 55Mn, 56Fe, 59Co, 60Ni, 63Cu, 66Zn, 75As, 82Se, 95Mo, 105Pd, 107Ag, 111Cd, 118Sn, 121Sb, 127I, 195Pt, 201Hg, 205Tl, 208Pb and 209Bi. Detailed analytical parameters are available elsewhere [10], [11]. These liquid samples were prepared for ICP-MS analysis by dilution 1/10 (v/v) with a solution containing HNO3 (1%), N-butanol (0.5%), Triton X-100 (0.1%) and two internal standards (rhodium (Rh) and indium (In) - 10ng/mL each). Batches were processed with a 6-points calibration curve and certified reference materials used as internal quality controls. Calibration solutions and internal standards were purchased from LabKings (Hilversum, The Netherlands), nitric acid and Triton X-100 from Merck (Darmstadt, Germany) and N-butanol from VWR Chemical (Rosny-sous-Bois, France). Certified reference materials

used were ClinChek® Controls (plasma and urine) and SRM 1869 (infant/adult nutritional formula II, milk/whey/soy-based), respectively bought from RECIPE (München, Germany) and NIST (Gaithersburg, MD, USA).

Note C. Relationship with ABO blood group

We investigated whether the signal observed in the *ABO* region is determinant of the ABO blood group and whether there exists a relationship between the ABO blood group type and zinc excretion levels.

<u>Genetics</u>

The ABO blood group can be primarily derived from two SNPs: rs8176746 and rs8176719 [12]. The rs8176719 deletion defines the O-blood group whereas the rs8176746 discriminates the A/B blood group as follows (adapted from Figure S2 d) from Melzer et al.):

**Genotype rs8176746 (B blood group SNP)**	**Genotype rs8176719 (O blood group SNP)**	**Haplotype (B-O)**	**Blood group phenotype**
G/G	del/del	G-del | G-del	O
G/G	del/C	G-del | G-C	A
G/G	C/C	G-C | G-C	A
G/T	del/del	G-del | T-del	O
T/T	del/del	T-del | T-del	O
T/T	del/C	T-del | T-C	B
T/T	C/C	T-C | T-C	B
G/T	del/C	G-del | T-C	B
G/T	C/C	G-C | T-C	AB

<u>Associations in the urinary zinc excretion GWAS:</u>

rs8176746 (T/G): pval = 0.73; rs8176719 (del/C): pval = 3.31e-23 → b(del)= -0.10

From this, we can conclude that the genetically determined O-group has a lower Zn excretion compared to the A/B group, but the A and B groups have similar Zn levels.

The r^2 between rs8176719 and the top SNP in the ABO region (rs2519093; pval = 4.63e-41) is 0.44.

Observational correlation

We further calculated the association between zinc levels and measured ABO blood group in the CoLaus cohort. ABO blood typing was available for 2,478 individuals with measured zinc levels and available covariates.

Tested model: Zn ~ isO adjusted for all relevant covariates (sex, age, BMI, smoking, batch)

Where "isO" defines whether a person has the O-group or not (i.e., has A, B, AB blood group phenotype)

The association between the O-group (N = 1,068) and zinc levels was highly significant: b_{obs} = -0.24; pval = 1.05e-9, corroborating that individuals with the O-blood type have lower zinc excretion.

Causal link between ABO blood type and Zn excretion

While genetically determined and measured blood type phenotypes are associated with zinc excretion, it remains possible that the regional causal SNP is in linkage disequilibrium with the two ABO blood type SNPs giving rise to these associations (hypothesis A) instead of the ABO blood type mediating the genetic association (hypothesis B) as illustrated in the following scheme:

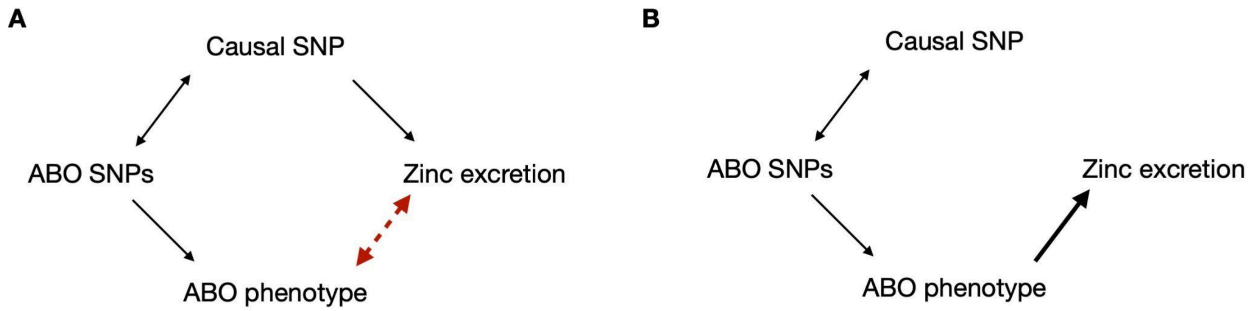

To test whether the hypothesis (A) or (B) is more likely, we first fitted a model where we regressed zinc levels on the regional ABO top SNP rs2519093 adjusted for all relevant covariates and PCs. We then regressed the residuals jointly on the measured blood type groups (O-group and B-group, where the B-group variable was defined as 1 for AB, 2 for B and 0 otherwise). The association p-values were 0.94 and 0.0422 for the B- and O-group, respectively. If the blood type group were independently associated with zinc levels (hypothesis B), we would expect a stronger association with zinc levels after adjusting for the regional top Zn. Thus, we believe that the linkage disequilibrium hypothesis (A) is more plausible.

Note D. Protocol and analyses for animal experiments

Dietary zinc supplementation

C57BL/6 mice were housed in a light- and temperature-controlled environment with *ad libitum* access to tap water and standard chow (Diet AO3, SAFE; 25/18 GR Mucedola Srl, Settimo Milanese, Italy). Mice (8-week old, males) were divided in three groups of 6 and fed either a diet containing ≤ 5 mg of Zn^{2+}/kg (zinc deficient diet, E15572-24 EF AIN93G, ssniff-Spezialdiäten-GmbH, Soest, Germany) or the same diet supplemented with either 30

mg/kg (Zn^{2+} control diet) or 300 mg/kg (Zn^{2+} excess diet) by addition of zinc chloride (229997, Sigma-Aldrich, Burlington, MA, USA). Mice were housed over-night in individual metabolic cages (UNO Roestvastaal BV, Zevenaar, The Netherlands) for urine and feces collection. Following euthanasia by sevoflurane (Attane, Piramal Healthcare Limited, Mumbai, India), blood was collected by venous puncture and centrifuged at 2000 g for 15 minutes at 4 °C in heparin-coated tubes (Sarstedt AG, Nürnbrecht, Germany) to allow separation of plasma from cells. Both kidneys were harvested and either snap-frozen in liquid nitrogen for RNA extraction, or further processed for histological analyses. Zinc concentration in the plasma, urine and feces was measured by inductively coupled plasma mass spectrometry (ICP-MS) [13].

Immunofluorescence analysis

Following collection, kidneys were fixed overnight at 4 °C in 4% formaldehyde (Sigma-Aldrich), dehydrated in increasing percentages of EtOH and subsequently embedded in paraffin. Paraffin blocks were cut into 5 μm-thick sections, deparaffinized in xylene and re-hydrated in decreasing ethanol concentrations. Heat mediated antigen retrieval was performed using 10 mM citrate buffer (pH 6.0) for 10 min at 98 °C in a Histos Pro Rapid Microwave Histoprocessor (Milestone Inc., Shelton, CT, USA). The sections were blocked for 30 min in PBS containing 3% BSA (Merck, Darmstadt, Germany), 30 mM glycine (Merck), 50 mM NH_4Cl (VWR international, Radnor, PA, USA) and 0.05% Tween-20 (Merck-Millipore) at room temperature and incubated with the primary antibody in a humidified chamber for either 1 hour at RT or overnight at 4 °C. The following primary antibodies were used: rabbit anti-ZnT2, A1195, BioVision, Milpitas, CA, USA; 1:300 for IF), sheep anti-megalin (kindly provided by P. Verroust and R. Kozyraki, INSERM, Paris, France; 1:1000 for IF). The sections were then incubated with the appropriate AlexaFluor-conjugated secondary antibody in PBS containing 1%

BSA for 2 hours at RT. (1:300, Life Technologies, Carlsbad, CA) for 1 hour at RT and counterstained with 1 μg Biotinylated Lotus Tetragonolobus Lectin (LTL; B-1325 Vector Laboratories) and 1 μM DAPI (D1306, Thermo Fischer Scientific). The sections were then washed, mounted using Prolong Gold Anti-fade reagent (P36930, Thermo Fisher Scientific) and viewed under a confocal microscope (Leica Microsystems GmbH, Wetzlar, Germany) using a ×63 1.4 NA oil immersion objective.

RNA extraction and RT-qPCR

Kidney microdissection was performed as previously described [13,14]. Analysis of gene expression levels in microdissected mouse kidney tubules was performed based on the MIQE guidelines [15]. Extraction of total RNA from tubules was performed using RNAqueous-Micro kit (Ambion, Huntingdon, UK), according to the manufacturer protocol. Contamination by genomic DNA was eliminated by DNase I treatment (Bio-Rad). Reverse transcriptase reaction with iScript™ cDNA Synthesis Kit (Bio-Rad) was executed with up to 1μg of RNA. The variations in mRNA levels of the target genes were established by relative RT-qPCR with a CFX96TM Real-Time PCR Detection System (Bio-Rad) and the iQ™ SYBR Green Supermix (Bio-Rad) for the detection of single PCR product accumulation. 100 nM of sense and anti-sense primers were used in a final volume of 20 μl in iQ™ SYBR Green Supermix (Bio-Rad) to perform RT-qPCR analyses (in duplicate). Primers specific to targets were designed with Beacon Design 2.0 (Premier Biosoft International, Palo Alto, CA, USA). PCR conditions were: 95 °C, 3 min followed by 40 cycles of 15 sec, 95 °C and 30 seconds at 60 °C. *Gapdh* was used as a reference housekeeping gene. The relative changes were determined by the formula: $2^{-\Delta\Delta ct}$ and expressed as fold change over the segment with the highest expression. Primer sequences are available in Table S3.

RNA-sequencing and bioinformatic analysis

RNA-sequencing of whole kidney lysates and bioinformatic analysis was performed at the Functional Genomics Center Zurich (FGCZ) of University of Zurich and ETH Zurich [16]. Sequencing was performed using an Illumina NovaSeq 6000 sequencer (Illumina, San Diego, CA, USA). Data were log_2-normalized and analyzed using the DESeq2 algorithm [17]. Volcano plots and overrepresentation analysis (ORA) scatterplots were generated with GraphPad Prism.

Supplementary Figures

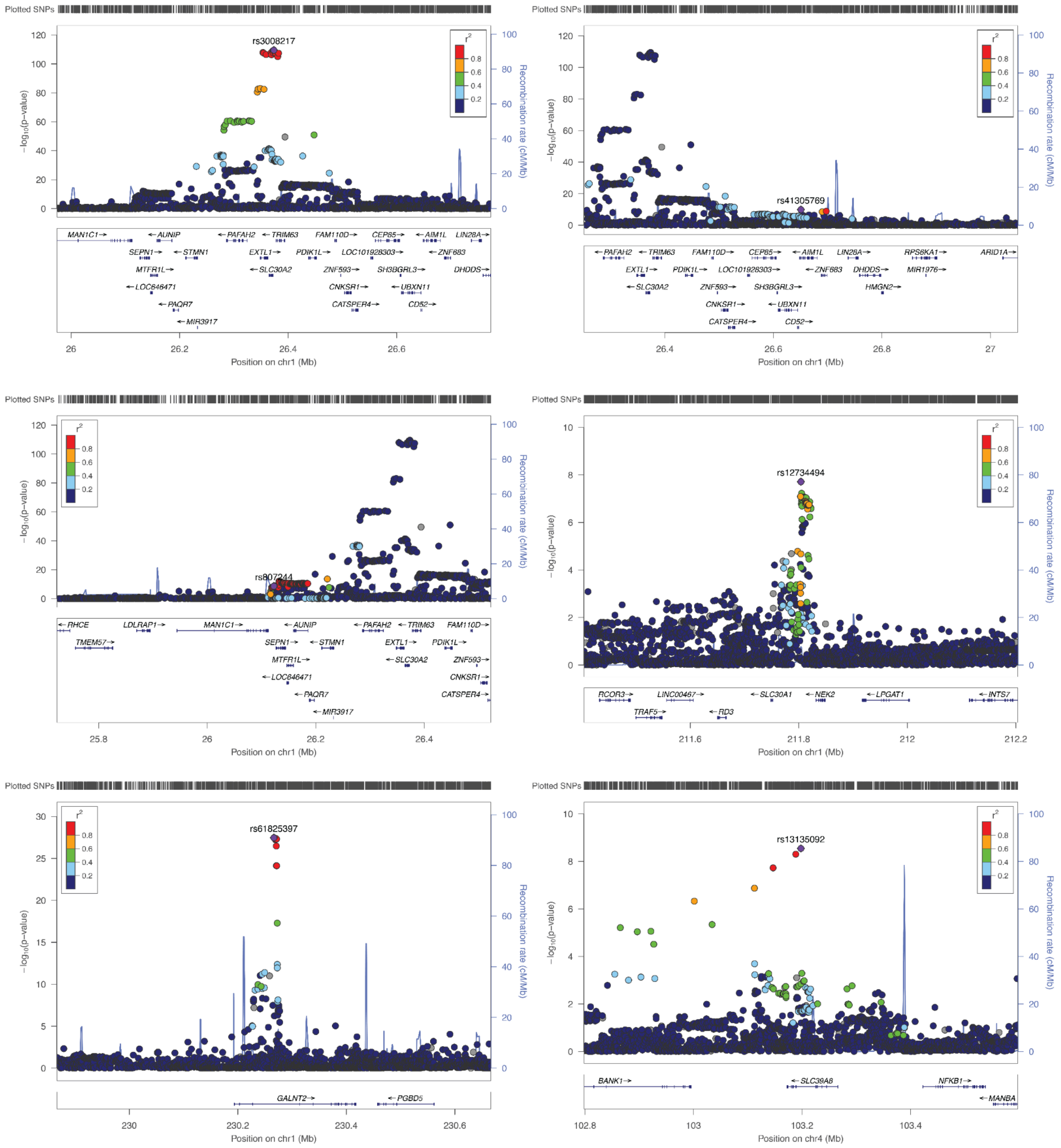

Figure A. Locus plots of the independent top hits.

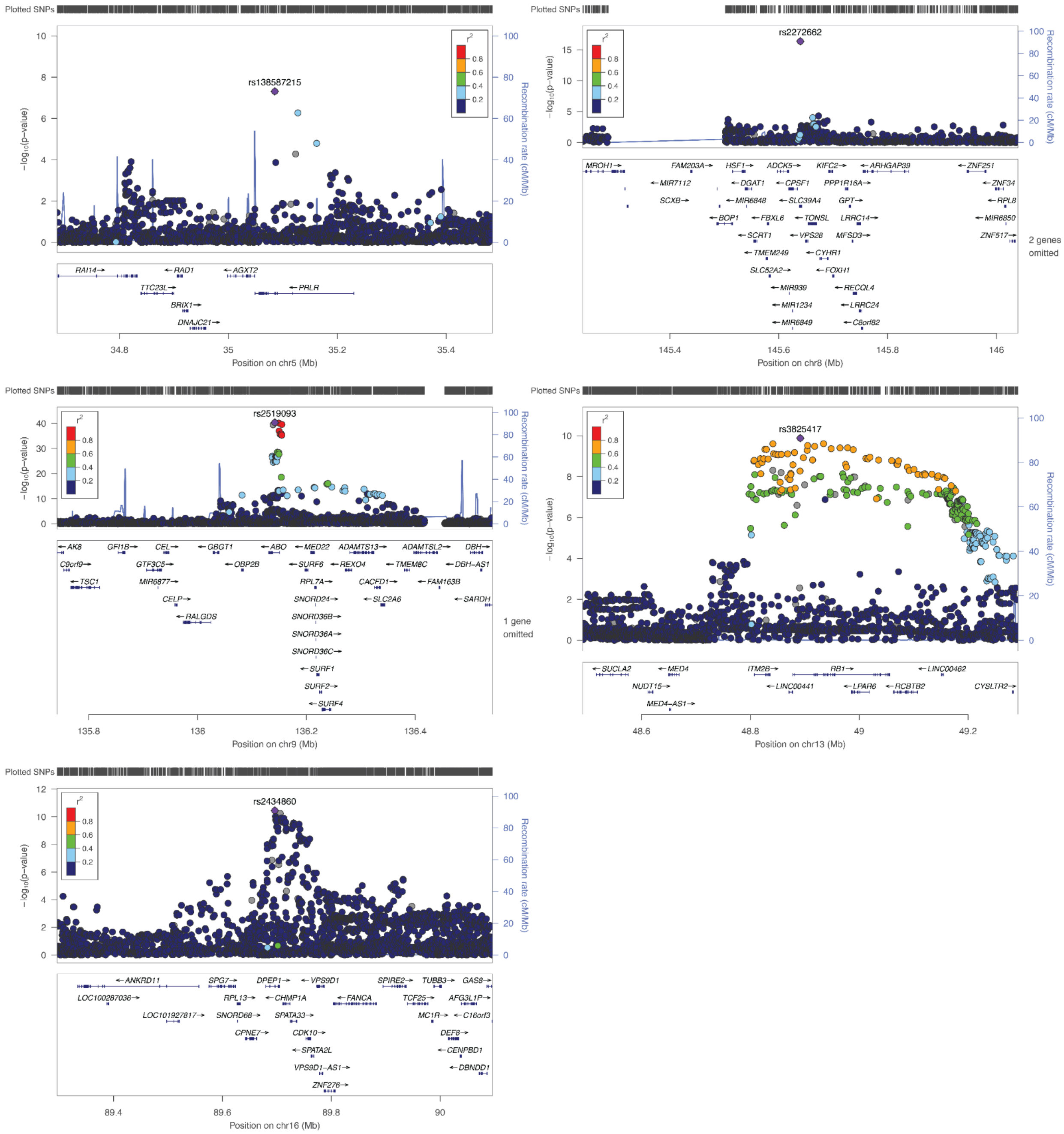

Figure B. Locus plots of the independent top hits.

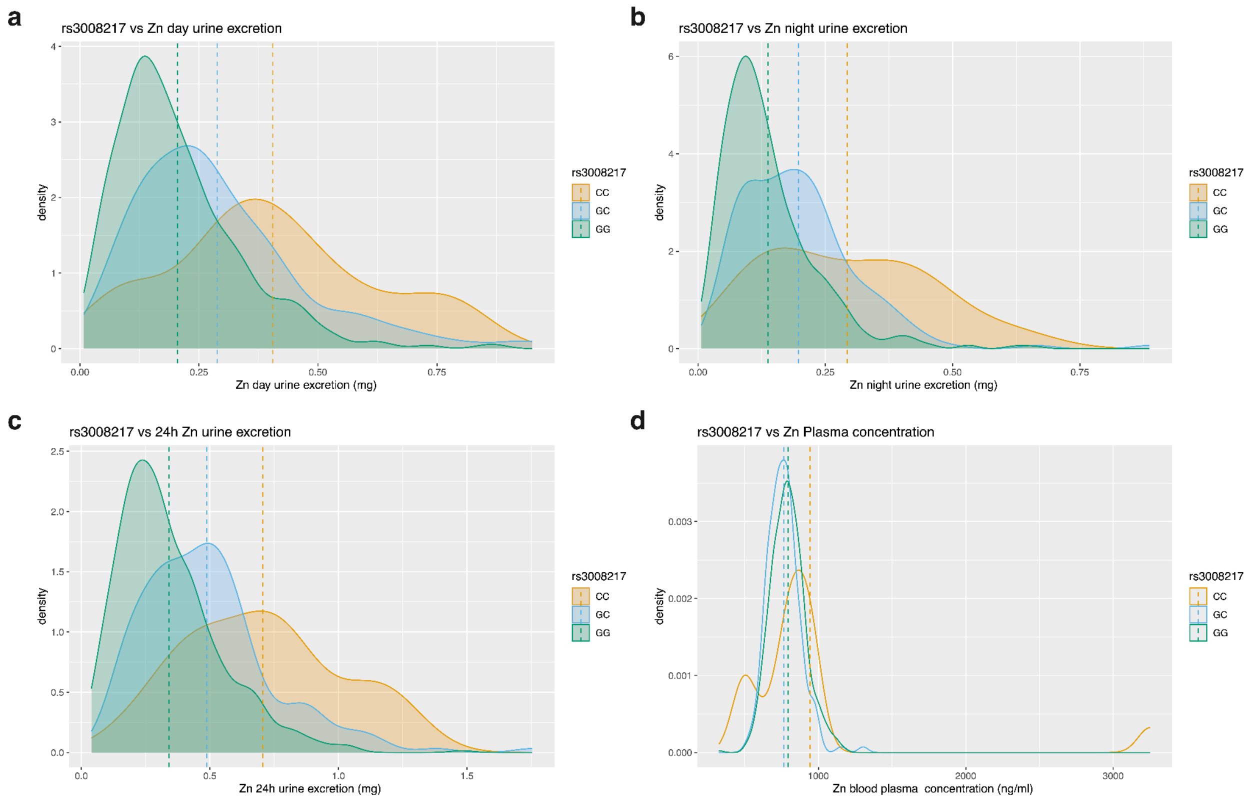

Figure C. Kernel-Density plots showing the distribution plasma concentration and urinary zinc excretion across rs3008217 genotypes in SKIPOGH.

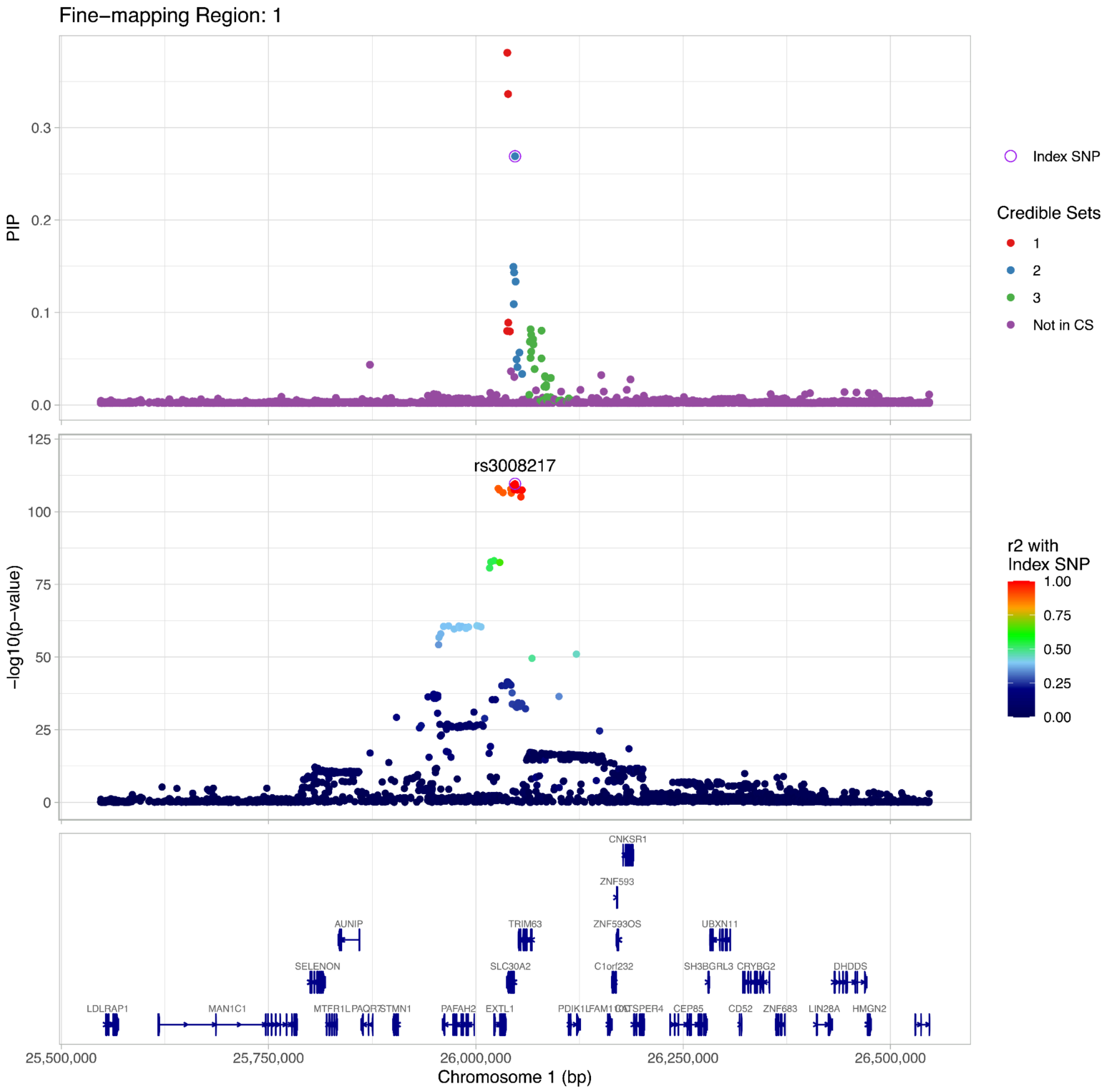

Figure D. Fine-mapping results of region 1.

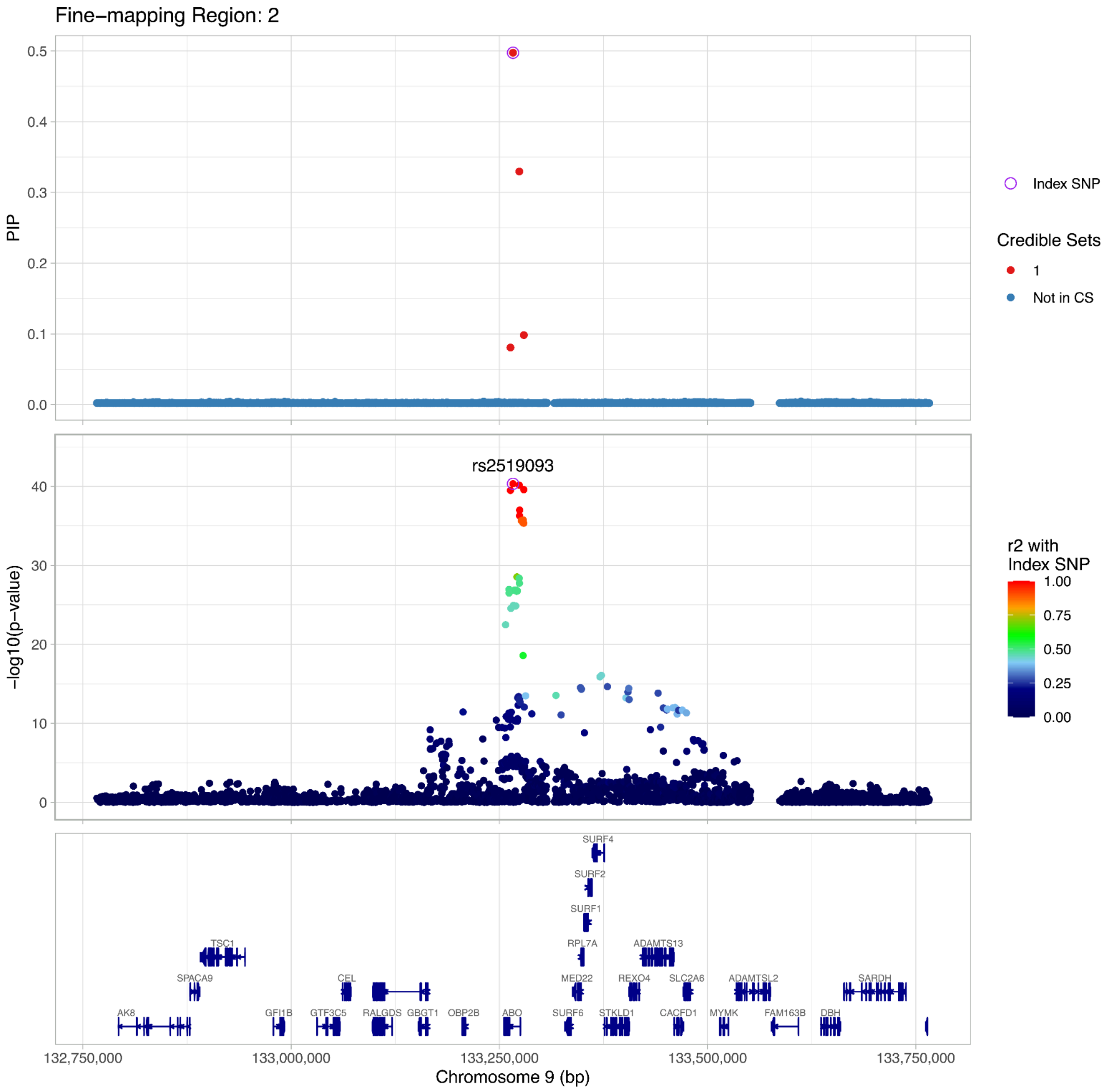

Figure E. Fine-mapping results of region 2.

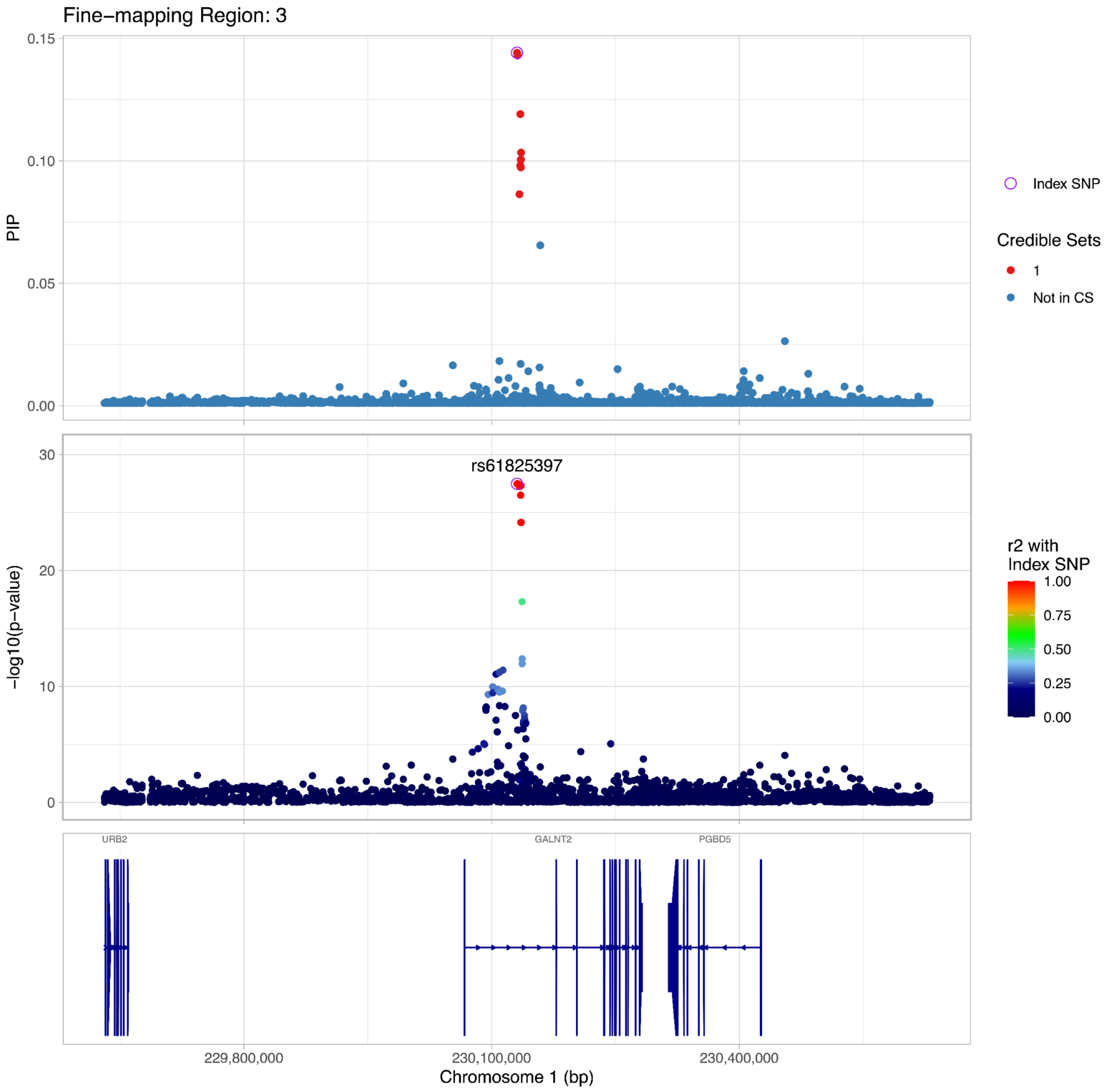

Figure F. Fine-mapping results of region 3.

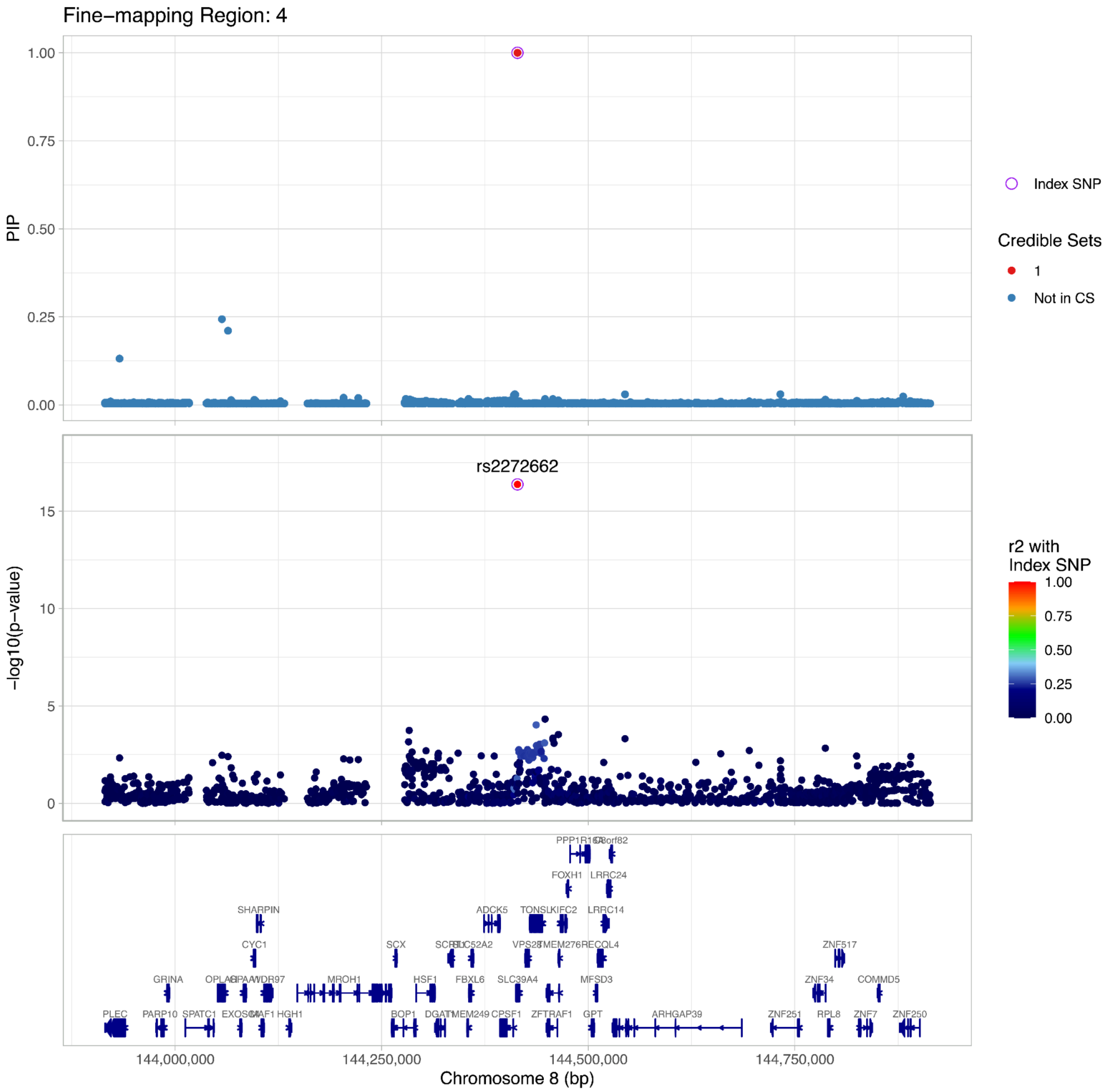

Figure G. Fine-mapping results of region 4.

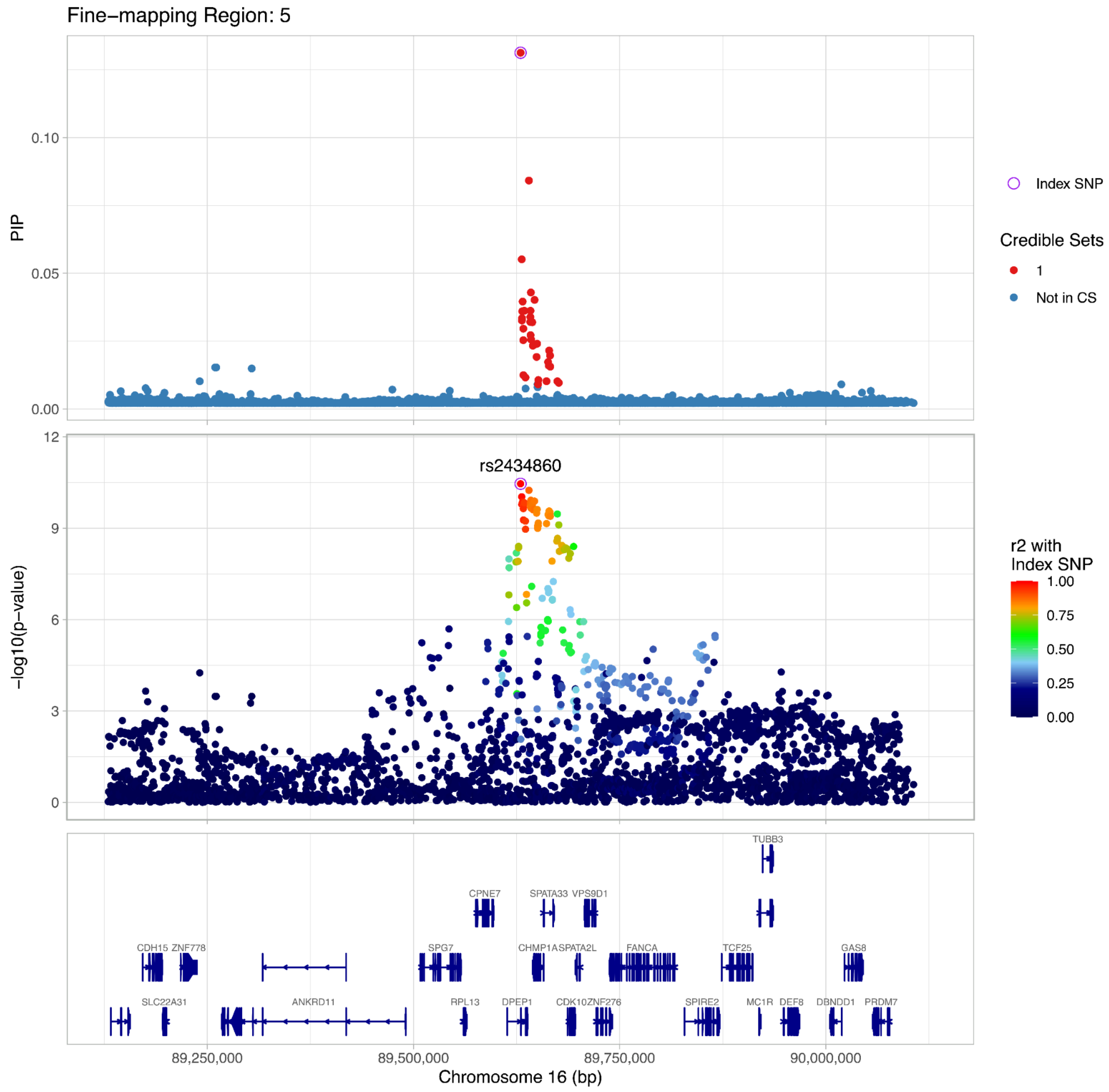

Figure H. Fine-mapping results of region 5.

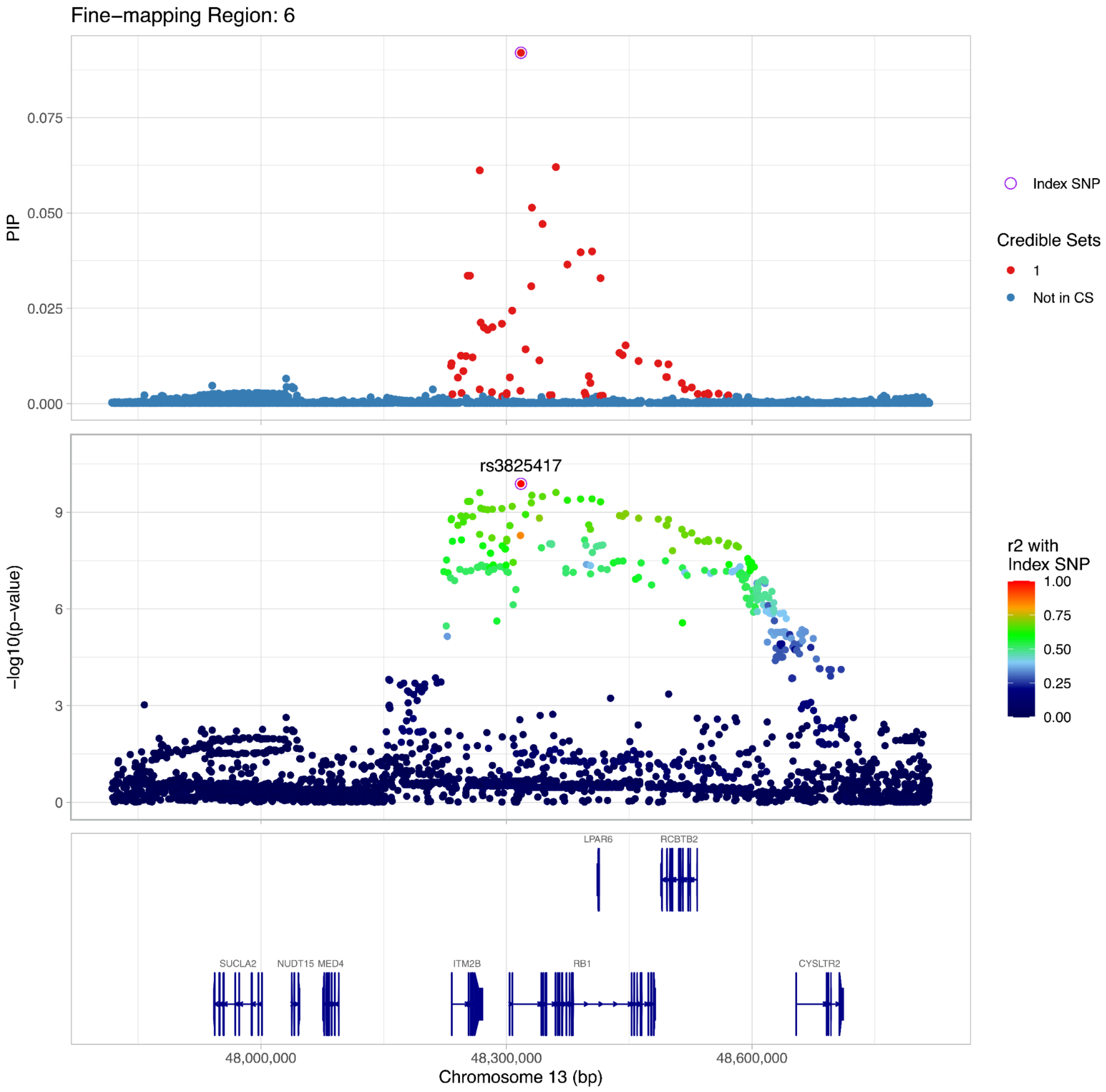

Figure I. Fine-mapping results of region 6.

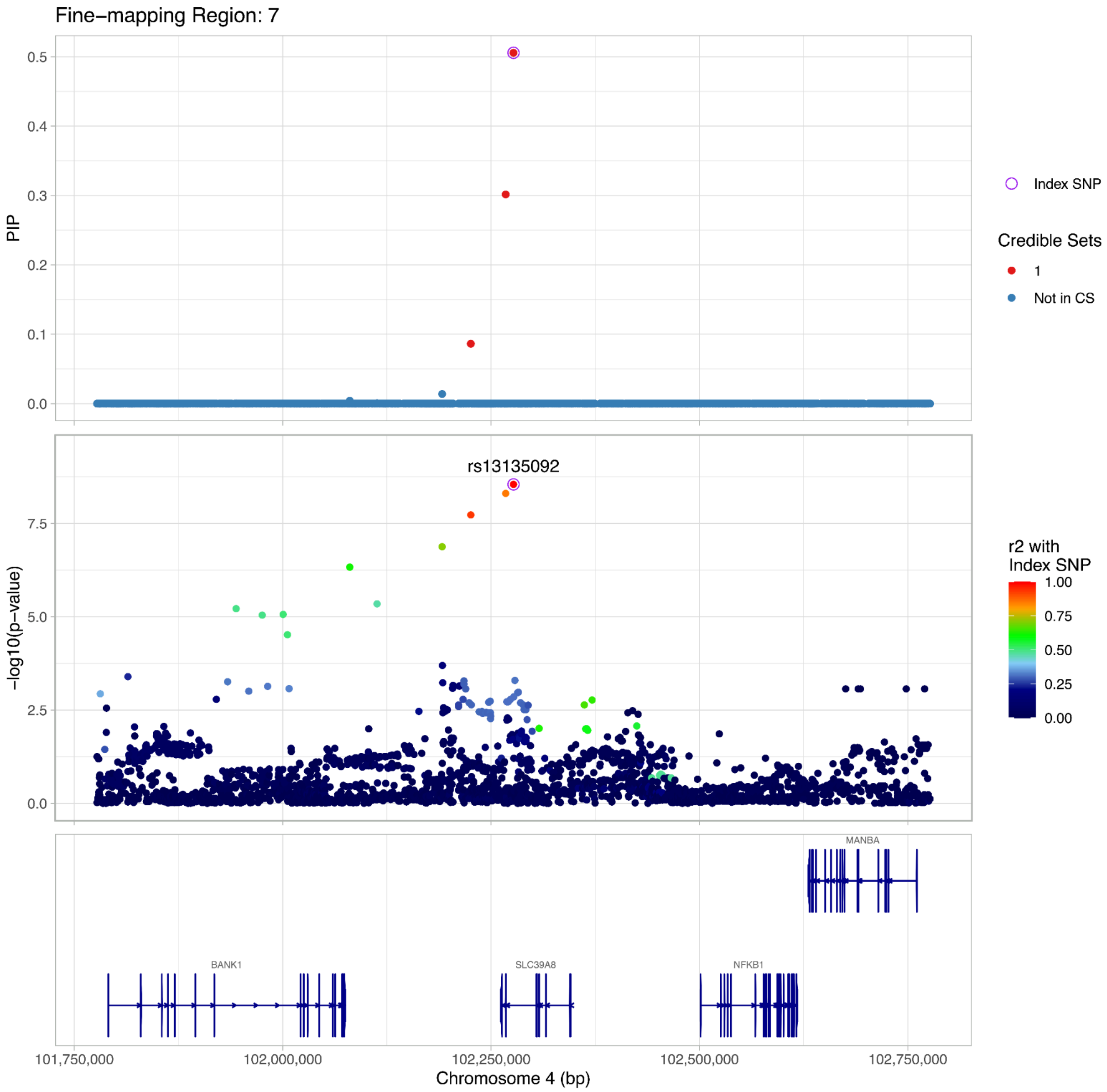

Figure J. Fine-mapping results of region 7.

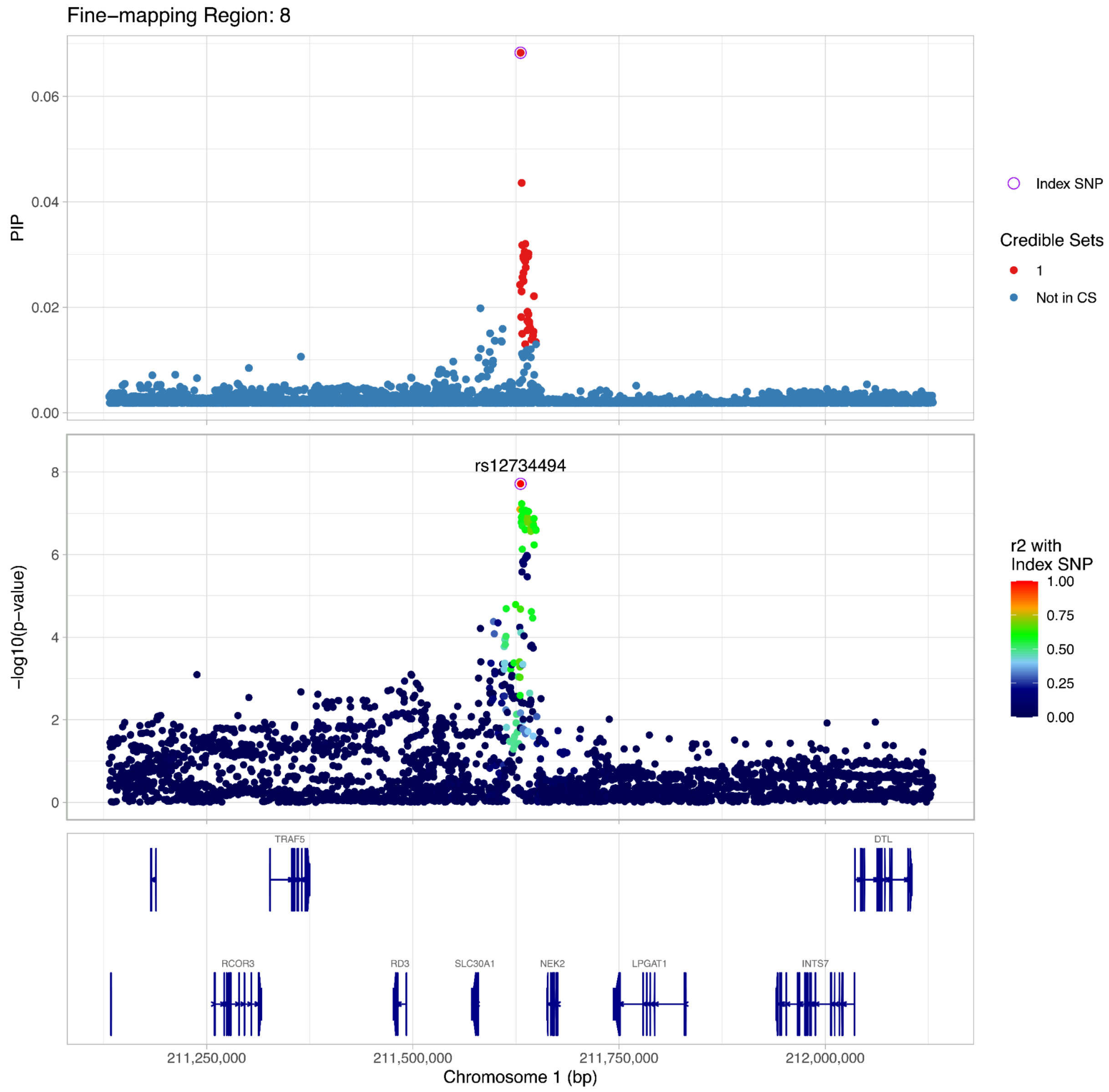

Figure K. Fine-mapping results of region 8.

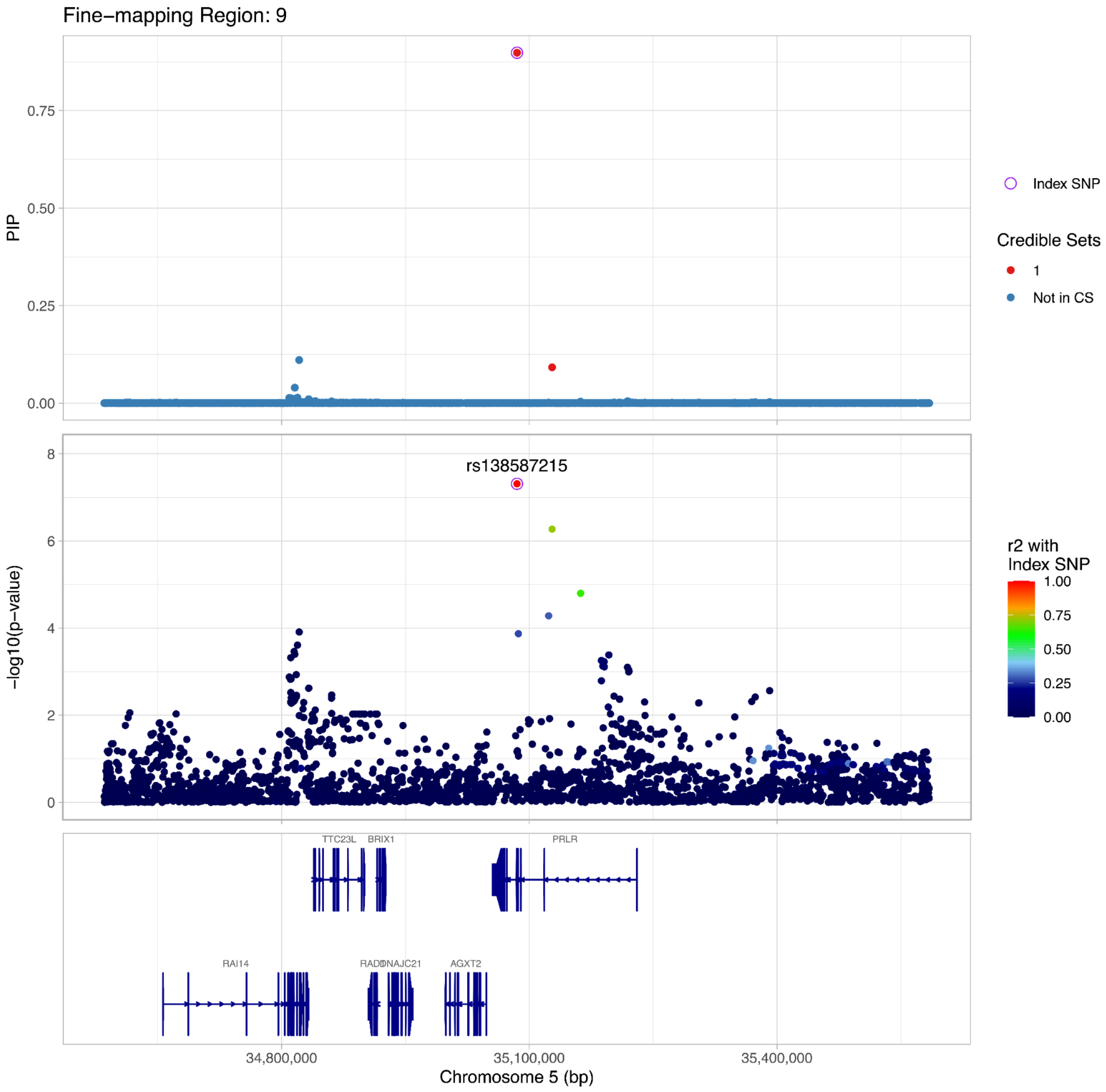

Figure L. Fine-mapping results of region 9.

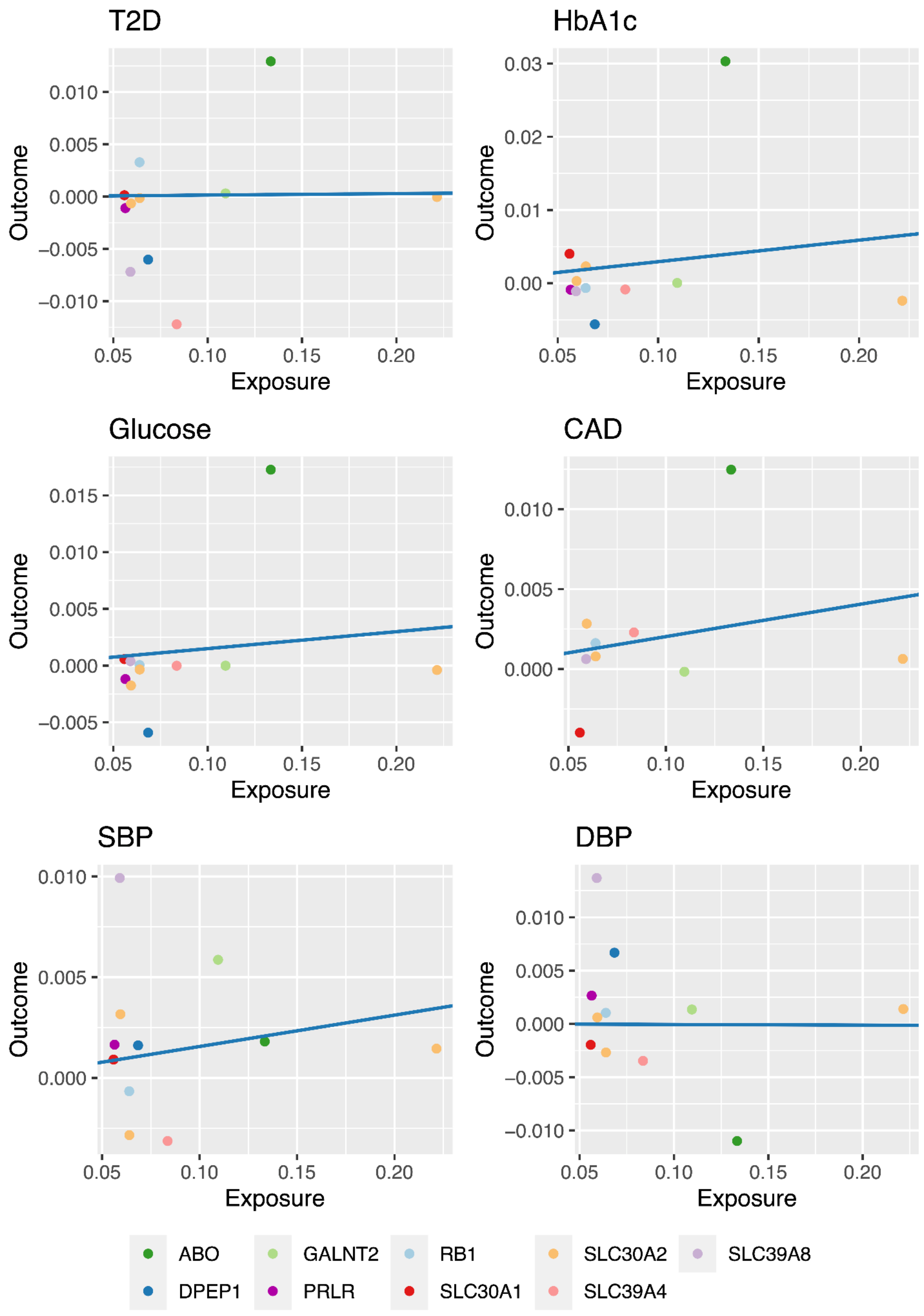

Figure M. Scatterplots of the exposure and outcome SNP associations of the instrumental variables (IVs) used in the forward Mendelian randomization analyses of zinc levels on traits.

IVs are colored by the gene they are mapping to. The line represents the inverse-variance weighted Mendelian randomization regression result.

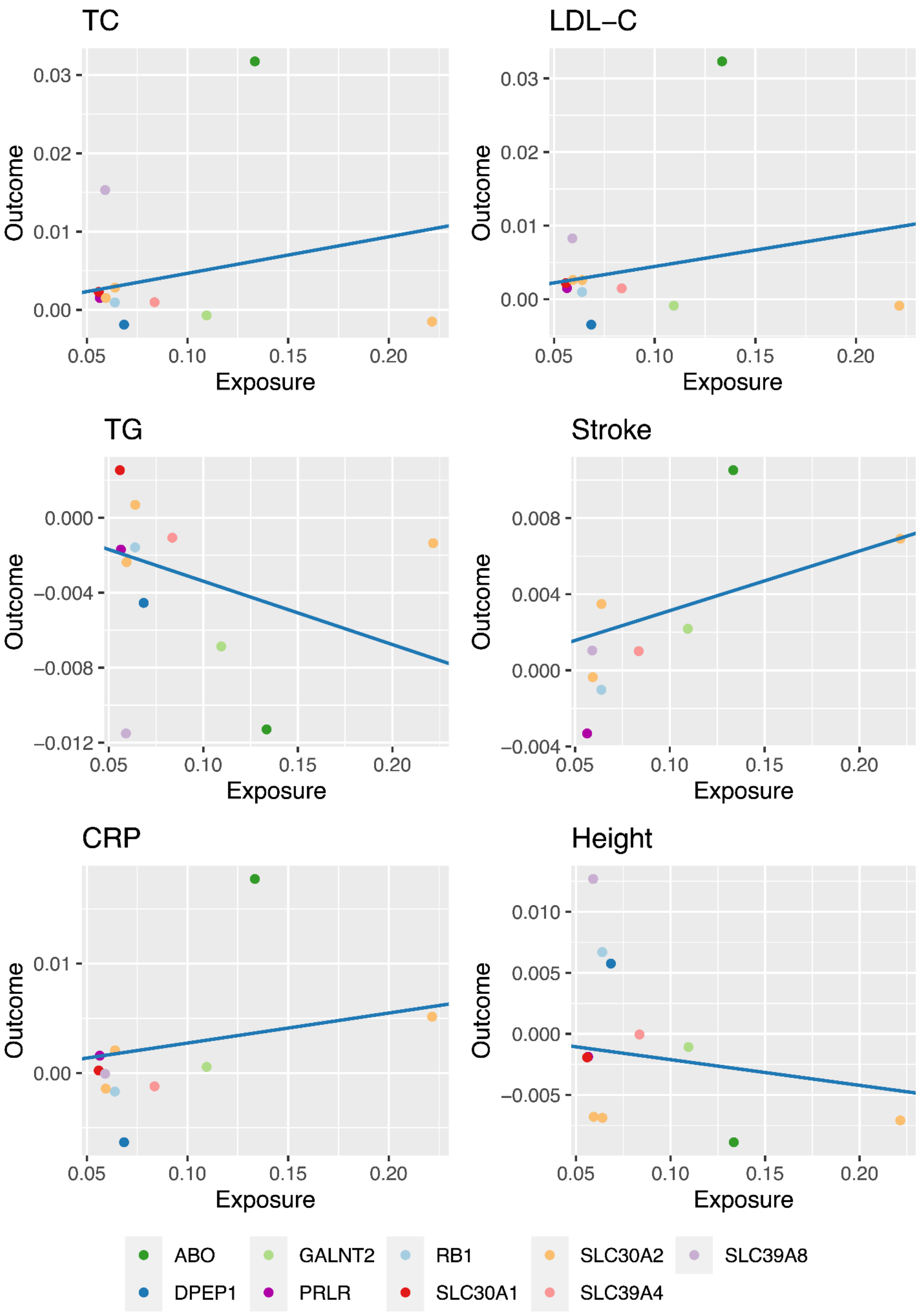

Figure N. Scatterplots of the exposure and outcome SNP associations of the instrumental variables (IVs) used in the forward Mendelian randomization analyses of zinc levels on traits.

IVs are colored by the gene they are mapping to. The line represents the inverse-variance weighted Mendelian randomization regression result.

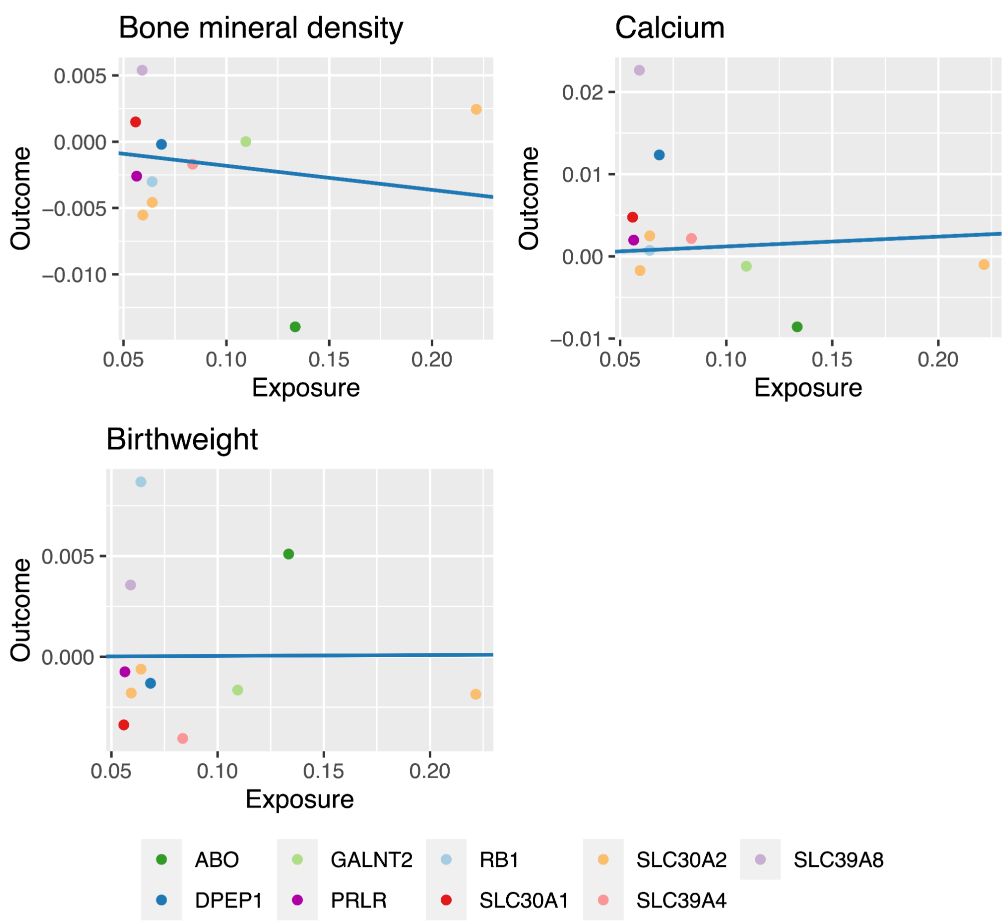

Figure O. Scatterplots of the exposure and outcome SNP associations of the instrumental variables (IVs) used in the forward Mendelian randomization analyses of zinc levels on traits.

IVs are colored by the gene they are mapping to. The line represents the inverse-variance weighted Mendelian randomization regression result.

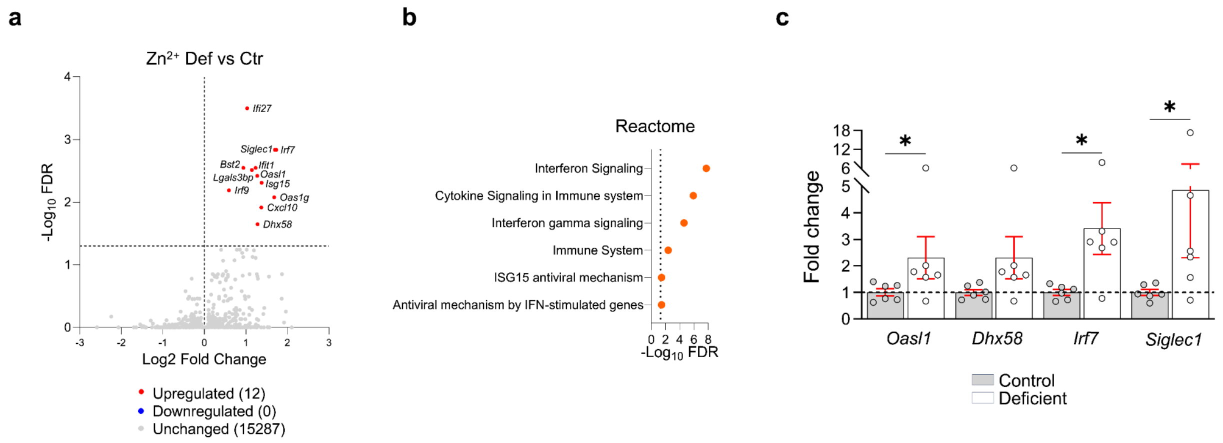

Figure P. RNA-seq analysis of Zn^{2+} deficient (Def) mouse kidneys compared to control (Ctr).

a Volcano plot differently expressed genes (DEGs) in Zn^{2+} Def kidneys compared to Ctr. Only 12 genes were found to be significantly upregulated in Zn^{2+} Def kidneys. In contrast, only three DEGs were found in Zn^{2+} Exc kidneys compared to Ctr (data not shown). **b** Over-representation analysis (ORA) showing activation of interferon signaling pathways in Zn^{2+} Def kidneys. **c** Validation of selected targets by RT–qPCR Zn^{2+} Def and Ctr kidneys (n = 6 animals per group). Values are relative to Zn^{2+} Ctr. Bars indicate the mean ± SEM. Mann-Whitney test: *$P < 0.05$.

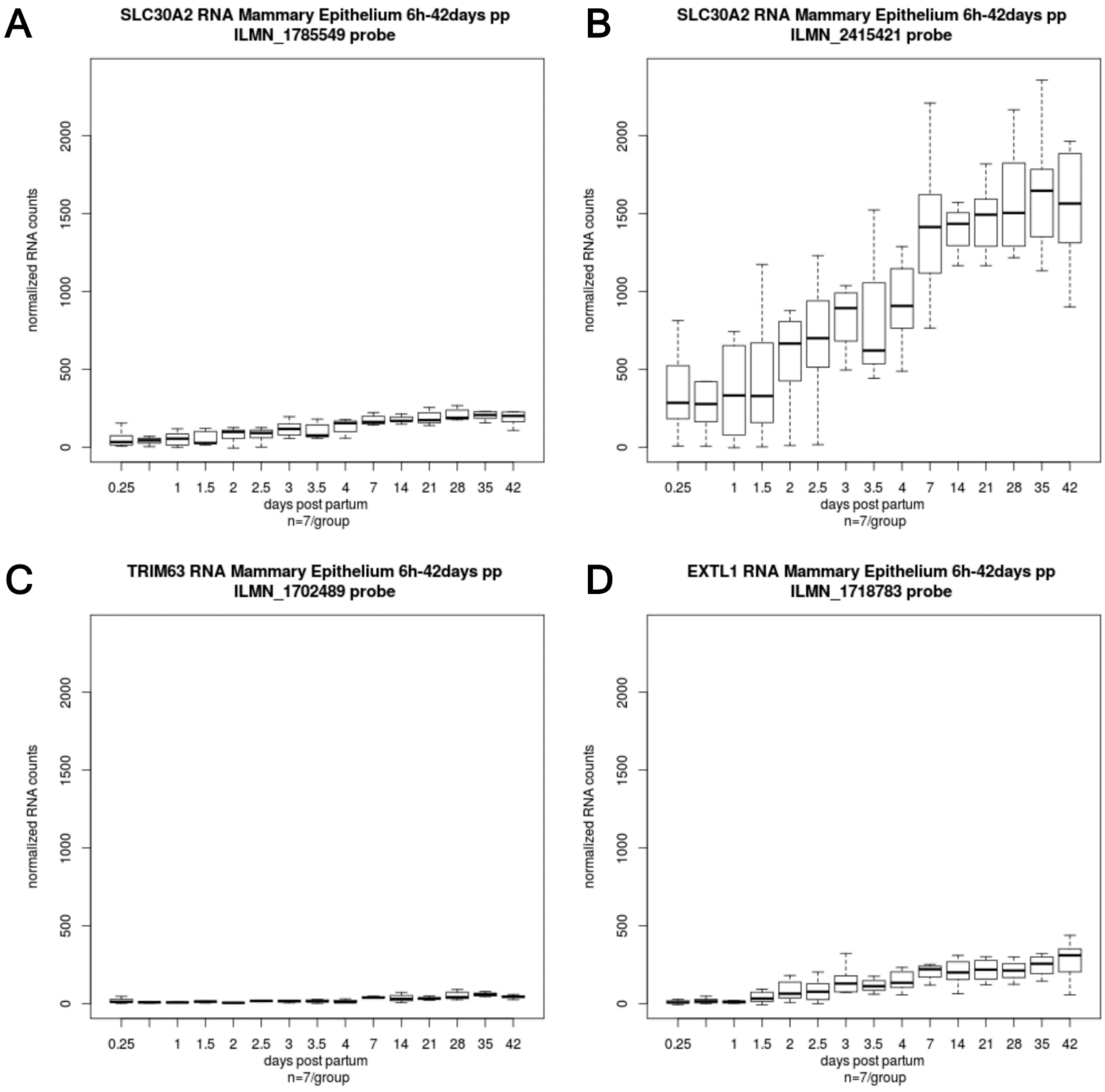

Figure Q. Gene expression levels in human mammary epithelium.
SLC30A2, *TRIM63*, and *EXTL1* gene expression levels (RNA) in human mammary epithelium tissue post-partum (Maningat et al., 2009 [18]).

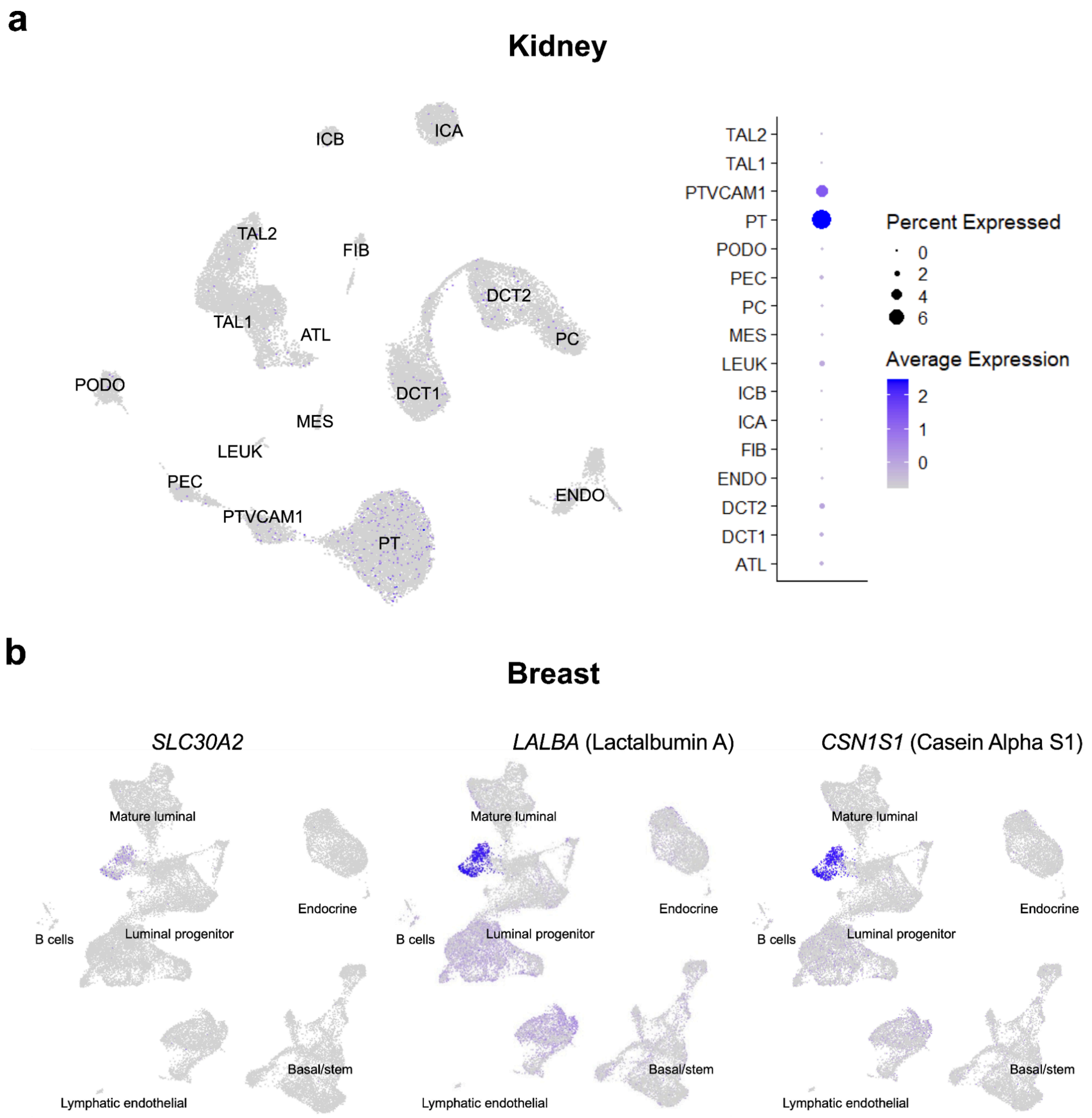

Figure R. scRNA-seq data in human kidney and breast.

a scRNA-seq data of human kidney, showing presence of SLC30A2 exclusively in proximal tubule cells. PCT, proximal convoluted tubule; PST, proximal straight tubule; PT_VCAM1, VCAM1(+) proximal tubule; PEC, parietal epithelial cells; ATL, ascending thin limb; TAL1, CLDN16(-) thick ascending limb; TAL2, CLDN16(+) thick ascending limb; DCT1, early distal convoluted tubule; DCT2, late distal convoluted tubule; PC, principal cells; ICA, type A intercalated cells; ICB, type B intercalated cells; PODO, podocytes; ENDO, endothelial cells; FIB, fibroblasts; MES, mesangial cells; LEUK, leukocytes. **b** scRNA-seq data of human healthy

breast tissue showing localized expression of *SLC30A2* in a subpopulation of luminal progenitor cells. Further analysis identified strong co-expression of the milk protein genes *LALBA* and *CSN1S1* in the same cell population. Data from Wilson et al., 2022 [19] and Bhat-Nakshatri et al.. 2021 [20].

Supplementary Tables

Table A: Urinary zinc excretion and blood zinc excretion genetic effects of the variants with genome-wide significance in either the urine or blood GWAS.

The urinary top hit rs3008217 was not available, and we used the proxy SNP rs3121763 ($r^2 = 1$). Likewise, we used the proxy SNP rs61578524 for rs61825397 (*GALNT2*, $r^2 = 1$) and the proxy SNP rs9316394 for rs7329065 (*RB1*/*RCBTB2*, $r^2 = 0.99$). For the blood Zn *CA1* hit rs10097917 we used the proxy SNP rs1496532 ($r^2 = 0.98$).

Chr	Position (GRCh37)	SNP	Mapped Gene	Effect allele	Other allele	Beta (urine)	p-value (urine)	Beta (blood)	p-value (blood)
1	199029020	rs322884	LINC01221	A	C	0.002	0.86	0.075	1.30E-09
8	86253982	rs1496532	CA1	T	C	-0.007	0.46	-0.119	7.66E-22
15	75355944	rs12591119	PPCDC; C15orf39	A	G	-0.005	0.60	0.161	1.44E-38
1	26122979	rs807244	SLC30A2	A	G	-0.059	2.36E-09	-0.004	0.74
1	26371788	rs3121763	SLC30A2	G	A	0.222	6.36E-110	-0.010	0.41
1	26650661	rs41305769	SLC30A2	A	G	-0.064	1.25E-10	-0.004	0.72
1	211803881	rs12734494	SLC30A1	A	G	0.056	1.92E-08	-0.006	0.62
1	230271567	rs61578524	GALNT2	A	G	-0.106	7.38E-25	-0.012	0.32
4	103198082	rs13135092	SLC39A8	A	G	0.059	2.84E-09	-0.033	8.05E-03
5	35085162	rs138587215	PRLR	T	C	0.056	4.87E-08	0.013	0.31
8	145639726	rs2272662	SLC39A4	T	C	0.084	4.20E-17	0.013	0.30
9	136141870	rs2519093	ABO	T	C	0.133	4.63E-41	-0.002	0.90
13	48891836	rs3825417	RB1/RCBTB2	A	G	-0.064	1.31E-10	-0.010	0.44
16	89696217	rs2434860	DPEP1/CHMP1A/ SPATA2L	T	C	-0.068	3.50E-11	0.025	4.22E-02

Table B. GWAS summary statistics used in the bidirectional Mendelian randomization analyses.

Disease/Trait	Abbreviation	Study/Consortium	Reference	Sample Size N (Ncases/controls)
Type 2 diabetes	T2D	DIAGRAM Consortium & UK Biobank	Mahajan et al., 2018 (https://doi.org/10.1038/s41588-018-0241-6)	74,124 / 824,006
HbA1c	HbA1c	UK Biobank Phenotype Code: 30750	http://www.nealelab.is/uk-biobank	344,182
Glucose	Glucose	UK Biobank Phenotype Code: 30740	http://www.nealelab.is/uk-biobank	314,916
C-reactive protein	CRP	UK Biobank Phenotype Code: 30710	http://www.nealelab.is/uk-biobank	343,524
Systolic blood pressure	SBP	UK Biobank Phenotype Code: 4080	http://www.nealelab.is/uk-biobank	408,211
Diastolic blood pressure	DBP	UK Biobank Phenotype Code: 4079	http://www.nealelab.is/uk-biobank	408,215
Total cholesterol	TC	UK Biobank Phenotype Code: 30690	http://www.nealelab.is/uk-biobank	344,278
Low-density lipoprotein cholesterol	LDL-C	UK Biobank Phenotype Code: 30780	http://www.nealelab.is/uk-biobank	343,621
Triglycerides	TG	UK Biobank Phenotype Code: 30870	http://www.nealelab.is/uk-biobank	343,992
Coronary artery disease	CAD	CARDIoGRAMplusC4D & UK Biobank	van der Harst et al., 2017 (https://doi.org/10.1161/CIRCRESAHA.117.312086)	122,733 / 424,528
Stroke	Stroke	MEGASTROKE Consortium	Malik et al., 2018 (https://doi.org/10.1038/s41588-018-0058-3)	40,585 / 406,111
Calcium	Calcium	UK Biobank Phenotype Code: 30680	http://www.nealelab.is/uk-biobank	315,153
Height	Height	UK Biobank Phenotype Code: 12144	http://www.nealelab.is/uk-biobank	360,388
Heel bone mineral density	Bone mineral density	UK Biobank Phenotype Code: 3148	http://www.nealelab.is/uk-biobank	206,496
Birth weight	Birthweight	UK Biobank Phenotype Code: 20022	http://www.nealelab.is/uk-biobank	205475

Table C. Mendelian randomization sensitivity analyses using the simple median and simple mode methods of forward causal effects of urinary zinc excretion levels on traits.

b_{MR} - MR causal effect; n_{IV} - number of instrumental variables.

Trait	Method	b_{MR} (forward)	se_{MR} (forward)	$pval_{MR}$ (forward)	n_{IV}
T2D	simple median	-0.002	0.013	8.63E-01	11
T2D	simple mode	-0.004	0.015	8.09E-01	11
T2D	MR Egger	-0.006	0.022	7.72E-01	11
HbA1c	simple median	-0.010	0.010	2.90E-01	11
HbA1c	simple mode	-0.011	0.011	3.55E-01	11
HbA1c	MR Egger	0.017	0.032	5.91E-01	11
Glucose	simple median	-0.000	0.009	9.83E-01	11
Glucose	simple mode	0.000	0.010	9.79E-01	11
Glucose	MR Egger	0.009	0.019	6.51E-01	11
CAD	simple median	0.012	0.010	2.36E-01	9
CAD	simple mode	0.011	0.011	3.38E-01	9
CAD	MR Egger	0.016	0.016	3.32E-01	9
SBP	simple median	0.016	0.010	1.05E-01	11
SBP	simple mode	0.019	0.013	1.88E-01	11
SBP	MR Egger	0.027	0.010	8.33E-03	11
DBP	simple median	0.010	0.010	3.04E-01	11
DBP	simple mode	-0.003	0.015	8.48E-01	11
DBP	MR Egger	0.017	0.018	3.28E-01	11
TC	simple median	0.026	0.013	4.92E-02	11
TC	simple mode	0.021	0.013	1.22E-01	11
TC	MR Egger	0.045	0.036	2.12E-01	11
LDL-C	simple median	0.027	0.013	3.20E-02	11
LDL-C	simple mode	0.030	0.013	4.26E-02	11
LDL-C	MR Egger	0.036	0.033	2.80E-01	11
TG	simple median	-0.030	0.013	2.46E-02	11
TG	simple mode	-0.028	0.019	1.74E-01	11
TG	MR Egger	-0.046	0.014	9.83E-04	11
Stroke	simple median	0.018	0.015	2.26E-01	9

Stroke	simple mode	0.015	0.021	4.84E-01	9
Stroke	MR Egger	0.033	0.013	1.02E-02	9
CRP	simple median	0.004	0.011	6.97E-01	11
CRP	simple mode	0.003	0.014	8.30E-01	11
CRP	MR Egger	0.020	0.019	2.89E-01	11
Height	simple median	-0.032	0.011	4.95E-03	11
Height	simple mode	-0.029	0.013	5.97E-02	11
Height	MR Egger	-0.004	0.019	8.37E-01	11
Bone mineral density	simple median	-0.020	0.016	1.93E-01	11
Bone mineral density	simple mode	-0.007	0.025	7.83E-01	11
Bone mineral density	MR Egger	-0.006	0.016	6.99E-01	11
Calcium	simple median	0.026	0.014	6.85E-02	11
Calcium	simple mode	0.012	0.013	3.75E-01	11
Calcium	MR Egger	0.032	0.027	2.40E-01	11
Birthweight	simple median	-0.013	0.012	2.78E-01	11
Birthweight	simple mode	-0.016	0.016	3.39E-01	11
Birthweight	MR Egger	0.000	0.014	9.90E-01	11

Table D. Sensitivity analyses of forward MR results of zinc on clinical phenotypes by leaving pleiotropic instrumental variables out.

"no SLC39A8": IV associated with SLC39A8 is left out, "no ABO": IV associated with ABO is left out, "no SLC39A8, no ABO": IVs associated with SLC39A8 and ABO are left out. b_{MR} – causal effect estimate; Q_{HET}pval: Cochran's heterogeneity Q-statistics p-value.

Trait	b_{MR} (no SLC39A8)	$pval_{MR}$ (no SLC39A8)	Q_{HET} pval (no SLC39A8)	b_{MR} (no ABO)	$pval_{MR}$ (no ABO)	Q_{HET} pval (no ABO)	b_{MR} (no SLC39A8, no ABO)	$pval_{MR}$ (no SLC39A8, no ABO)	Q_{HET} pval (no SLC39A8, no ABO)
T2D	0.007	7.62E-01	1.51E-14	-0.018	3.41E-01	3.66E-08	-0.013	4.77E-01	7.48E-07
HbA1c	0.036	3.20E-01	1.21E-59	-0.008	3.97E-01	1.22E-02	-0.008	4.68E-01	6.73E-03
Glucose	0.020	3.38E-01	1.82E-16	-0.005	4.70E-01	4.81E-01	-0.005	4.46E-01	3.83E-01
CAD	0.019	2.03E-01	9.46E-10	0.002	7.16E-01	2.05E-01	0.002	7.79E-01	1.41E-01
SBP	0.009	3.30E-01	1.04E-02	0.015	3.50E-01	4.80E-08	0.008	4.73E-01	5.78E-03
DBP	-0.011	5.04E-01	7.30E-11	0.015	4.69E-01	5.40E-15	0.005	6.77E-01	5.78E-04
TC	0.043	2.24E-01	3.03E-59	0.011	6.07E-01	1.71E-15	-0.001	9.31E-01	3.23E-01
LDL-C	0.044	2.22E-01	1.27E-61	0.006	6.48E-01	5.16E-05	-0.000	9.69E-01	1.80E-01
TG	-0.029	3.48E-02	4.46E-07	-0.024	1.58E-01	1.34E-09	-0.016	1.37E-01	4.96E-03
Stroke	0.035	1.20E-03	1.42E-01	0.024	8.51E-03	7.67E-01	0.025	9.17E-03	6.54E-01
CRP	0.032	1.25E-01	1.94E-18	0.009	4.16E-01	2.51E-03	0.009	4.38E-01	1.26E-03
Height	-0.027	1.03E-01	8.59E-12	-0.008	7.38E-01	1.16E-22	-0.018	3.10E-01	8.15E-10
Bone mineral density	-0.019	2.71E-01	6.41E-07	0.004	7.07E-01	4.73E-02	0.000	9.83E-01	2.03E-01
Calcium	0.000	9.94E-01	4.34E-15	0.031	3.55E-01	5.49E-39	0.015	4.43E-01	7.52E-10
Birthweight	0.000	9.84E-01	2.11E-04	-0.005	7.17E-01	7.92E-04	-0.008	5.82E-01	1.39E-03

Table E. Mendelian randomization sensitivity analyses using the simple median and simple mode methods of reverse causal effects of traits on urinary zinc excretion levels.

b_{MR} - MR causal effect; n_{IV} - number of instrumental variables.

Trait	Method	b_{MR} (reverse)	se_{MR} (reverse)	$pval_{MR}$ (reverse)	n_{IV}
T2D	simple median	0.144	0.055	8.57E-03	315
T2D	simple mode	0.128	0.170	4.55E-01	315
T2D	MR Egger	0.135	0.038	3.27E-04	315
HbA1c	simple median	0.001	0.041	9.80E-01	733
HbA1c	simple mode	0.147	0.141	2.99E-01	733
HbA1c	MR Egger	-0.022	0.026	4.13E-01	733
Glucose	simple median	0.089	0.081	2.68E-01	168
Glucose	simple mode	0.123	0.199	5.36E-01	168
Glucose	MR Egger	0.056	0.052	2.80E-01	168
CAD	simple median	0.076	0.079	3.38E-01	226
CAD	simple mode	0.090	0.223	6.87E-01	226
CAD	MR Egger	0.013	0.054	8.16E-01	226
SBP	simple median	0.064	0.069	3.54E-01	374
SBP	simple mode	-0.055	0.245	8.22E-01	374
SBP	MR Egger	0.074	0.046	1.06E-01	374
DBP	simple median	0.169	0.073	2.09E-02	319
DBP	simple mode	0.039	0.310	8.99E-01	319
DBP	MR Egger	0.139	0.054	9.90E-03	319
TC	simple median	0.011	0.048	8.20E-01	391
TC	simple mode	0.102	0.125	4.15E-01	391
TC	MR Egger	0.008	0.032	7.95E-01	391
LDL-C	simple median	0.039	0.051	4.42E-01	368
LDL-C	simple mode	0.121	0.111	2.76E-01	368
LDL-C	MR Egger	-0.015	0.032	6.40E-01	368
TG	simple median	0.125	0.041	2.67E-03	591
TG	simple mode	0.236	0.101	1.95E-02	591
TG	MR Egger	0.139	0.026	7.87E-08	591
Stroke	simple median	-0.483	0.245	4.87E-02	11

Stroke	simple mode	-0.364	0.415	4.01E-01	11
Stroke	MR Egger	-0.319	0.204	1.18E-01	11
CRP	simple median	-0.020	0.048	6.82E-01	488
CRP	simple mode	0.119	0.141	3.98E-01	488
CRP	MR Egger	-0.053	0.029	6.33E-02	488
Height	simple median	0.010	0.023	6.50E-01	2294
Height	simple mode	0.035	0.097	7.15E-01	2294
Height	MR Egger	0.013	0.015	3.70E-01	2294
Bone mineral density	simple median	0.040	0.036	2.62E-01	581
Bone mineral density	simple mode	0.040	0.115	7.25E-01	581
Bone mineral density	MR Egger	0.039	0.022	8.14E-02	581
Calcium	simple median	0.118	0.052	2.25E-02	415
Calcium	simple mode	0.103	0.161	5.22E-01	415
Calcium	MR Egger	0.084	0.033	1.09E-02	415
Birthweight	simple median	-0.023	0.086	7.89E-01	126
Birthweight	simple mode	-0.205	0.235	3.85E-01	126
Birthweight	MR Egger	-0.027	0.067	6.82E-01	126

Table F. Pearson correlations between allele frequencies (frequency of urinary zinc-increasing allele) and zinc deficiency prevalences.

Measured zinc deficiency prevalences stem from 13 countries and estimated zinc deficiency prevalences from 82 countries. R corresponds to the Pearson correlation; and p-values to one-sided Pearson correlation tests.

SNP	R (est. Zn prev.)	P-value (est. Zn prev.)	R (meas. Zn prev.)	P-value (meas. Zn prev.)
rs807244 (SLC30A2)	-0.143	9.01E-01	0.159	3.02E-01
rs3008217 (SLC30A2)	0.332	1.14E-03	0.079	3.98E-01
rs41305769 (SLC30A2)	-0.015	5.53E-01	-0.062	5.79E-01
rs12734494 (SLC30A1)	-0.136	8.88E-01	0.245	2.10E-01
rs61825397 (GALNT2)	-0.061	7.08E-01	0.074	4.05E-01
rs13135092 (SLC39A8)	0.474	3.50E-06	0.235	2.19E-01
rs138587215 (PRLR)	-0.451	1.00E+00	-0.423	9.25E-01
rs2272662 (SLC39A4)	0.394	1.24E-04	0.308	1.53E-01
rs2519093 (ABO)	-0.458	1.00E+00	-0.221	7.66E-01
rs3825417 (RB1/RCBTB2)	0.459	7.09E-06	0.123	3.45E-01
rs2434860 (DPEP1)	0.413	5.66E-05	0.123	3.44E-01

Table G. Extract from the GWAS atlas PheWAS database for rs3008217.

All traits with p-values < 5E-06 are listed. Available online at: https://atlas.ctglab.nl/, accessed OCT 2024.

atlas ID	PMID	Year	Domain	Trait	P-value	N	EA	NEA
3187	31427789	2019	Skeletal	Standing height	4.745E-11	385748	G	C
4043	30124842	2018	Skeletal	Height	1E-10	693529	G	C
3471	31427789	2019	Metabolic	Impedance measures - Trunk predicted mass	7.823eE-09	379469	G	C
3470	31427789	2019	Metabolic	Impedance measures - Trunk fat-free mass	9.755E-09	379507	G	C
3462	31427789	2019	Metabolic	Impedance measures - Arm fat-free mass (right)	1.222E-08	379723	G	C
3463	31427789	2019	Metabolic	Impedance measures - Arm predicted mass (right)	1.459E-08	379716	G	C
3444	31427789	2019	Metabolic	Impedance measures - Whole body water mass	1.666E-08	379835	G	C
3443	31427789	2019	Metabolic	Impedance measures - Whole body fat-free mass	1.856E-08	379804	G	C
3446	31427789	2019	Metabolic	Impedance measures - Basal metabolic rate	4.142E-08	379821	G	C
3412	31427789	2019	Skeletal	Sitting height	9.041E-08	385393	G	C
3467	31427789	2019	Metabolic	Impedance measures - Arm predicted mass (left)	1.434E-07	379638	G	C
3458	31427789	2019	Metabolic	Impedance measures - Leg fat-free mass (left)	2.097E-07	379766	G	C
3459	31427789	2019	Metabolic	Impedance measures - Leg predicted mass (left)	2.374E-07	379761	G	C
3466	31427789	2019	Metabolic	Impedance measures - Arm fat-free mass (left)	3.706E-07	379653	G	C
3455	31427789	2019	Metabolic	Impedance measures - Leg predicted mass (right)	0.000001217	379793	G	C
3454	31427789	2019	Metabolic	Impedance measures - Leg fat-free mass (right)	0.000001437	379793	G	C

INSPIRE Consortium - Author information

Janet E. Williams[1], Brenda M. Murdoch[1], Gloria E. Otoo[2], Samwel Mbugua[3], Elizabeth W. Kamau-Mbuthia[3], Egidioh W. Kamundia[3], Debela K. Gindola[4], Juan M. Rodríguez[5], Rossina G. Pareja[6], Daniel W. Sellen[7], Sophie E. Moore[8,9], Andrew M. Prentice[9], James A. Foster[10], Linda J. Kvis[11], Lars Bode[12,13], Mark A. McGuire[14], Michelle K. McGuire[15], Courtney L. Meehan[16]

1. Animal, Veterinary and Food Sciences, University of Idaho, USA
2. Department of Nutrition & Food Science, University of Ghana, Accra, Ghana
3. Department of Human Nutrition, Egerton University, Nakuru, Kenya
4. Department of Anthropology, Hawassa University, Hawassa, Ethiopia
5. Department of Nutrition and Food Science, Complutense University of Madrid, Madrid, Spain
6. Nutrition Research Institute, Lima, Peru
7. Department of Anthropology, University of Toronto, Toronto, Ontario, Canada
8. Department of Women and Children's Health, King's College London, London, UK
9. MRC Unit The Gambia at the London School of Hygiene and Tropical Medicine, Fajara, Gambia
10. Department of Biological Sciences, University of Idaho, Moscow, Idaho, USA
11. Faculty of Medicine, Lund University, Lund, Sweden
12. Larsson-Rosenquist Foundation Mother-Milk-Infant Center of Research Excellence, University of California, San Diego, La Jolla, California, USA
13. Department of Pediatrics, University of California, San Diego, La Jolla, California, USA
14. Department of Animal, Veterinary and Food Sciences, University of Idaho, Moscow, Idaho, USA
15. Margaret Ritchie School of Family and Consumer Sciences, University of Idaho, Moscow, Idaho, USA
16. Department of Anthropology, Washington State University, Pullman, Washington, USA

References

1. Ha N-T, Freytag S, Bickeboeller H. Coverage and efficiency in current SNP chips. Eur J Hum Genet. 2014;22: 1124–1130.

2. Blauwendraat C, Faghri F, Pihlstrom L, Geiger JT, Elbaz A, Lesage S, et al. NeuroChip, an updated version of the NeuroX genotyping platform to rapidly screen for variants associated with neurological diseases. Neurobiol Aging. 2017;57: 247.e9–247.e13.

3. Reed E, Nunez S, Kulp D, Qian J, Reilly MP, Foulkes AS. A guide to genome-wide association analysis and post-analytic interrogation. Stat Med. 2015;34: 3769–3792.

4. McCarthy S, Das S, Kretzschmar W, Delaneau O, Wood AR, Teumer A, et al. A reference panel of 64,976 haplotypes for genotype imputation. Nat Genet. 2016;48: 1279–1283.

5. Hor H, Kutalik Z, Dauvilliers Y, Valsesia A, Lammers GJ, Donjacour CEHM, et al. Genome-wide association study identifies new HLA class II haplotypes strongly protective against narcolepsy. Nat Genet. 2010;42: 786–789.

6. Das S, Forer L, Schönherr S, Sidore C, Locke AE, Kwong A, et al. Next-generation genotype imputation service and methods. Nat Genet. 2016;48: 1284–1287.

7. Loh P-R, Danecek P, Palamara PF, Fuchsberger C, A Reshef Y, K Finucane H, et al. Reference-based phasing using the Haplotype Reference Consortium panel. Nature Genetics. 2016;48: 1443–1448.

8. Fuchsberger C, Abecasis GR, Hinds DA. minimac2: faster genotype imputation. Bioinformatics. 2015;31: 782–784.

9. Schlosser P, Scherer N, Grundner-Culemann F, Monteiro-Martins S, Haug S, Steinbrenner I, et al. Genetic studies of paired metabolomes reveal enzymatic and transport processes at the interface of plasma and urine. Nat Genet. 2023;55: 995–1008.

10. Perrais M, Trächsel B, Lenglet S, Pruijm M, Ponte B, Vogt B, et al. Reference values for plasma and urine trace elements in a Swiss population-based cohort. Clinical Chemistry and Laboratory Medicine (CCLM). 2024;62: 2242–2255.

11. Perrais M, Thomas A, Augsburger M, Lenglet S. Comparison of Dried Blood Spot and Microtube Techniques for Trace Element Quantification by ICP-MS. J Anal Toxicol. 2023;47: 175–181.

12. Melzer D, Perry JRB, Hernandez D, Corsi A-M, Stevens K, Rafferty I, et al. A genome-wide association study identifies protein quantitative trait loci (pQTLs). PLoS Genet. 2008;4: e1000072.

13. Lauer E, Villa M, Jotterand M, Vilarino R, Bollmann M, Michaud K, et al. Imaging mass spectrometry of elements in forensic cases by LA-ICP-MS. Int J Legal Med. 2017;131: 497–500.

14. Glaudemans B, Terryn S, Gölz N, Brunati M, Cattaneo A, Bachi A, et al. A primary culture system of mouse thick ascending limb cells with preserved function and uromodulin processing. Pflugers Arch. 2014;466: 343–356.

15. Bustin SA, Benes V, Garson JA, Hellemans J, Huggett J, Kubista M, et al. The MIQE guidelines: minimum information for publication of quantitative real-time PCR experiments. Clin Chem. 2009;55: 611–622.

16. Hatakeyama M, Opitz L, Russo G, Qi W, Schlapbach R, Rehrauer H. SUSHI: an exquisite recipe for fully documented, reproducible and reusable NGS data analysis. BMC Bioinformatics. 2016;17: 228.

17. Love MI, Huber W, Anders S. Moderated estimation of fold change and dispersion for RNA-seq data with DESeq2. Genome Biol. 2014;15: 550.

18. Maningat PD, Sen P, Rijnkels M, Sunehag AL, Hadsell DL, Bray M, et al. Gene expression in the human mammary epithelium during lactation: the milk fat globule transcriptome. Physiol Genomics. 2009;37: 12–22.

19. Wilson PC, Muto Y, Wu H, Karihaloo A, Waikar SS, Humphreys BD. Multimodal single cell sequencing implicates chromatin accessibility and genetic background in diabetic kidney disease progression. Nat Commun. 2022;13: 5253.

20. Bhat-Nakshatri P, Gao H, Sheng L, McGuire PC, Xuei X, Wan J, et al. A single-cell atlas of the healthy breast tissues reveals clinically relevant clusters of breast epithelial cells. Cell Rep Med. 2021;2: 100219.
