# Supplementary material for: Genetic determinants of zinc homeostasis and its role in cardiometabolic diseases
Source: PLoS Genet. 2025 Dec 1;21(12):e1011928. doi: 10.1371/journal.pgen.1011928 (PMC12677789; doi:10.1371/journal.pgen.1011928)
Supplement: Attachment — Submitted filename: Rebuttal.pdf [file pgen.1011928.s008.pdf]

Colour code:

Answers to the reviewers are written in green.

Unchanged elements borrowed from the manuscript are written in dark blue (italic),

and changes in the manuscript are written in brown (italic).

Response letter

Reviewer #1: The research detailed in this paper is an impressive and comprehensive investigation into the genetic architecture of urinary zinc levels in individuals of European-ancestry and would be of interest to those in the fields of genetic medicine, public health and population genetics. Sadler et al. highlight the role of of SLC30A2 in the metabolism of zinc and provide valuable insights into how zinc levels may be maintained when dietary levels are deficient. They also attempt to evaluate the genetic risk of individual populations to zinc deficiency given the limited data available.

The manuscript is well-written and presents convincing data but can be improved in terms of 1) greater context of the results and 2) discussion of genetic variation of the SLC30A2 locus at the population level. I have outlined my specific, minor comments below.

We thank the reviewer for the positive assessment of our work and the comments aimed at improving the clarity and the discussion of the results in a greater context.

1) Zinc is a trace metal micronutrient, meaning that it cannot be synthesised by the diet and instead its levels are completely determined by the diet. It may be beneficial to the reader to understand that deficiencies of zinc (and other micronutrients) may then be caused by either a) malnutrition/poor diet or b) low levels of zinc in soils where dietary foodstuffs are grown. When discussing evolutionary arguments (see comments 4 - 7), this is a key point.

We agree with the reviewer to add more context on the origin of zinc in the body

While zinc is ubiquitously present in the human habitat, it is only available for human metabolism via dietary intake. Its main dietary sources are meat, fish, dairy, legumes, and whole grain cereals, with a greater bioavailability in animal products than in plant-based foods. Phytate compounds in plants tend to chelate zinc and reduce its absorption in the human gut [1,5,7,8]. Histidine and methionine, as well as EDTA and organic acids on the other hand increase its absorption [https://doi.org/10.1093/jn/130.5.1378S]. Zinc content in food is directly linked to zinc content in agricultural soils. Several studies have highlighted the relationship between dietary zinc, nutritional zinc status and soil zinc content [https://doi.org/10.1007/bf01088441, https://doi.org/10.3390/nu11020221, https://doi.org/10.1038/s41598-021-88304-6].

Through malabsorption, malnutrition or increased losses, zinc deficiency leads to serious health conditions including growth stunting, hypogonadism, impaired immune response, skin lesions, and neurocognitive disorders [9].

2) At the bottom of page 6: “whilst circulating zinc levels are physiologically important, they are not a reliable indicator of individual zinc status” – please explain why this is

We agree that this statement lacks context and we have updated the section as follows:

While circulating zinc levels are physiologically important, they are not a reliable indicator of individual zinc status [16]. Plasma zinc, recognized as the most reliable and widely utilized biomarker of population zinc status, is sensitive to severe zinc deficiency; however, it has limited capacity to detect mild to moderate deficiency states [https://doi.org/10.3389/fnut.2022.1057156]. Although previous studies suggested that plasma zinc increases with dietary supplementation [https://doi.org/10.3945/jn.110.122812, https://doi.org/10.1007/s12011-020-02163-2], the overall picture is more complex. An analysis of NHANES data shows that zinc concentrations are more related to sex, age, serum albumin and hemoglobin levels as well as the time of blood draw than to dietary zinc intake or zinc supplements [https://doi.org/10.1093/jn/nxy105]. Studies assessing zinc enrichment in largely consumed food also show no change in plasma zinc concentration despite improvement in selected growth indicators in children [https://doi.org/10.1093/ajcn/nqab379, https://doi.org/10.3390/nu17071137]

Zinc is involved in numerous essential cellular processes affecting gene expression, enzymatic functions and structural aspects in all compartments of the human body [https://doi.org/10.3390/nu12030762]. These findings are supported by data showing that only about 1.5% of body zinc is found in the blood compared with bone (36.7%) and muscle tissue (49.5%), where zinc is bioavailable but not readily mobilizable [https://doi.org/10.3390/nu12030762].

3) They suggest that the body maintains zinc levels by adaptations of the renal handling. The authors may consider comparing this the hierarchy of selenium supply to various organs in cases of selenium deficiency, e.g., referencing work such as Sarangi et al, 2018.

We agree that adding context for the homeostatic response of the body to varying zinc levels is useful and have updated the section as follows:

Our experimental investigations in a murine model of zinc feeding support this observation by showing large increases in urine but not blood zinc levels upon zinc feeding, suggesting that the body maintains stable circulating zinc levels by regulating fecal excretion and adapting its renal handling through urinary elimination, in addition to modulating intestinal absorption. These findings are important considering that there is currently no truly reliable biomarker of zinc status [33, https://doi.org/10.3389/fnut.2022.1057156]. This adaptation is supported by other data showing variability of type and location of zinc transporters depending on zinc status. For example, zinc deficiency promotes accumulation of ZIP4 on the surface membrane of intestinal enterocytes [https://doi.org/10.1016/j.jbc.2021.100320]. High extracellular zinc on the other hand triggers lysosomal removal of ZIP4 [https://doi.org/10.1515/bc.2007.149]. Another buffering and regulatory mechanism for zinc are the metallothioneins. They bind zinc intracellularly and serve as a dynamic pool for stabilizing zinc levels [https://doi.org/10.1007/s12011-017-1119-7].

4) There has been a wide body of work exploring genetic adaptation of zinc transporters across global human populations (e.g., Zhang et al., 2015; Roca-Umbert et al. 2022; Engelken et al., 2014) that should be referenced in this manuscript. This literature includes strong evidence of local adaptation in SLC39A8 and SLC39A4 mentioned in the manuscript and should be included.

We agree that examples of genetic adaptations should be included in the discussion of the findings of ecological correlations we have updated the text as follows

While higher zinc excretion in human milk may be beneficial for the offspring, elevated urinary zinc excretion in regions with zinc-deficient diets can be detrimental for health. For other zinc transporters, SLC39A8 and SLC39A4, genetic adaptation has already been demonstrated [https://doi.org/10.1038/srep09658, https://doi.org/10.1371/journal.pgen.1010950, https://doi.org/10.1371/journal.pgen.1004128]. In a similar fashion, *it is likely that an optimal level of zinc excretion exists for SLC30A2, which has likewise been shaped by genetic adaptation and deviations of urinary excretion in either direction may be disadvantageous to human health. Therefore, future research should examine a possibly non-linear (U-shaped) relationship between urinary zinc concentration and phenotypic outcomes.*

5) There is some confusion / lack of clarity of the hypotheses described in the "ecological correlation at the SLC30A2 locus" section. The stated hypothesis is that regions with higher frequencies of alleles that increase urinary zinc will be found in regions with higher zinc deficiency. They find only a significant positive correlation between allele frequency and estimated zinc deficiency – this is simply a correlation between allele frequency and dietary habits. For the hypothesis to be true, the authors would have to show a positive correlation between explicitly measured zinc levels (although, I recognise that data is a) limited and b) blood data that the authors show cannot be expected to represent urinary data). It should be clearer throughout this section that the correlation discussed is not reflecting metabolic response to zinc and may simply be reflecting a correlation between ancestry and culture/dietary practices/socio-economic factors.

We agree that the result section "Ecological correlation at the SLC30A2 locus" lacked some clarity, and we have updated the section to highlight the limitations early on, and to provide a clearer interpretation of the results. The updated section reads as follows:

*The SNPs identified in the GWAS on urinary zinc levels exhibit large geographical variation in allele frequencies across countries, as do global variations in zinc deficiency prevalence. Since the discovered variants have a rather substantial effect on urinary zinc levels, we hypothesised that regions with higher urinary zinc-increasing allele frequencies may exhibit higher zinc deficiency. Given the available data, this hypothesis could only be tested indirectly using dietary-based zinc deficiency estimates and limited measured serum zinc data, which may not reflect renal zinc handling. To test this hypothesis, we analyzed country-specific allele frequencies and zinc deficiency data. Notable differences in per-country allele frequencies were observed for genetic variants in zinc transporters including rs2272662 (SLC39A4, 33.1%-94.4%), rs807244 (SLC30A2, 1.4%-35.3%), rs3008217 (SLC30A2, 5.3%-43.7%), rs12734494 (SLC30A1, 12.8%-56.1%) as well as for rs2434860 (DPEP1, 36.8%-99.6%) and rs3825417 (RB1/RCBTB2, 14.8%-88.0%; Figure 5**a**, Dataset S6). We then correlated the frequency of urinary zinc-increasing alleles with measured and estimated per-country zinc deficiency prevalences, for which data on 13 and 82 countries, respectively, were available. Measured zinc deficiency data came from a review that assessed plasma/serum zinc concentrations from national surveys in low-and middle-income countries [27]. Estimated zinc deficiency prevalences were derived from dietary zinc intake using national food balance sheets, making them more reflective of nutritional factors [1,28,29]. Note that, based on the 13 complete observations, measured and estimated zinc deficiency prevalences were positively correlated (Pearson correlation = 0.46; one-sided p-value = 0.05). Furthermore, we found significant positive correlations with*

*estimated zinc deficiency prevalence (p-value < 0.05) for five of the eleven GWAS SNPs, including variants in SLC39A4, SLC30A8, SLC30A2, DPEP1, and RB1/RCBTB2 (Figure 5**a**, Table S6), supporting an overall trend that zinc-increasing alleles correlate positively with estimated zinc deficiency (one-sided binomial test p-value = 1.12E-04). However, as estimated zinc deficiency prevalence is derived from dietary intake data, these associations may primarily reflect population/ancestry differences in diet, or socio-economic factors rather than direct metabolic responses to the lack of zinc. On the other hand, no significant correlations were observed for measured zinc deficiency prevalence, and any apparent trends (8 positive vs 3 negative). However, this lack of evidence for a correlation should be interpreted cautiously given the very small sample size and the lack of overlap between the genetic basis of urinary and serum zinc measures.*

*Next, we aggregated the allele frequencies of the eleven SNPs into a per-country genetic risk score (GRS), calculated by multiplying allele frequencies by their effect sizes on urinary zinc levels. Across 82 countries, the GRS was highest in sub-Saharan Africa (Figure 5**b**, Dataset S6), a region also bearing the highest burden of zinc deficiency. A significant positive correlation was observed between the GRS and estimated zinc deficiency prevalence (Pearson correlation = 0.4, p-value = 2E-04), while a positive but non-significant correlation was found with measured zinc deficiency prevalence (Pearson correlation = 0.21, p-value = 0.5; Figure 5**c**). Although these results are interesting, their interpretation is very difficult and we provide some hypotheses in the Discussion."*

We rewrote the respective text in the Discussion:

"Since the SLC30A2 locus has by far the largest impact on urinary zinc levels and the colocalization signal was the clearest for this gene, we focussed on SLC30A2 and its lead variant (rs3008217). Our ecological correlation analysis showed significant positive correlation between estimated zinc deficiency prevalence and the frequency of the rs3008217-C allele. Its frequency varied considerably across human populations, displaying high allele frequencies in Sub-Saharan Africa (≥40%) [1, 30]. Considering that the SLC30A2/ZnT2 transporter is responsible for zinc secretion in human milk, we hypothesized that this correlation may be induced by a possible selection mechanism for offspring survival in regions with elevated prevalence of zinc deficiency, with the rs3008217-C allele leading to an increased zinc secretion in human milk, and thus to greater zinc amounts available for the breastfed child [10]. Alternatively, the frequency of the rs3008217 C allele may have dropped as a consequence of the out-of-Africa bottleneck event. However, the GWAS for human milk zinc concentration in an African sample found a non-significant association of the rs3008217-C allele with zinc levels in human milk, and despite the modest sample size, the effect - even if there is one - is likely to be substantially attenuated compared to that in urine. The hypothesis of natural selection via maternal milk zinc excretion is supported by PheWAS data in European samples, reporting the rs3008217-C (higher urinary zinc excretion) allele being associated with decreased height, trunk, arm, leg, body mass, as well as poorer forced vital capacity (S7 Table in File S1).

Interestingly, the ecological correlation seems to hold not only for the SLC30A2 variant, but more globally for the polygenic score for high zinc excretion. Which might suggest that geographic distribution of genetic determinants driving high urinary zinc may contribute to the observed geographic patterns of zinc deficiency. If the genetic basis of zinc excretion is

similar between breast milk and urine, the above-outlined breast milk hypothesis can provide an alternative explanation. Regardless of the underlying causes, populations with inadequate dietary zinc and phytate intake or inadequate intestinal zinc absorption also carry an increased genetic risk for high urinary zinc levels, potentially exacerbating existing nutritional deficiencies. Despite the proposed hypotheses involving natural selection, bottleneck event or adaptation, the observed ecological correlations may well be confounded and simply reflect shared geographic patterns in genetic variation, dietary practices, and socio-economic context.

We also mention the uncertainty about the presented hypotheses in the limitation section of the Discussion:

"Fifth, while the correlation between zinc-increasing allele frequency and zinc deficiency aligns with expectations, the ecological correlation analysis was only borderline conclusive. We believe this is likely due to power limitations: the number of countries with country-specific zinc deficiency estimates is limited, these estimates are likely to be noisy, and the genetic alleles examined are relatively weak predictors of zinc excretion, especially since the polygenic predictor we used is based on European samples only. Furthermore, other potential mechanisms can also give rise to such geographic correlations amongst populations, including different ancestral zinc levels in soils, distinct ancestral diet, divergent socio-economic conditions or differences in pathogen-induced zinc starvation. More in-depth analysis separating these effects necessitates future research, as was conducted for selenium [76], but it is out of the scope of our current investigation.

6) It may also be interesting to calculate the correlation between the measured and estimated zinc deficiency data (with the caveat that zinc levels in the blood may not be informative on zinc deficiency). Deviations between measured zinc levels and estimated zinc levels may indicate differences in metabolism of zinc amongst populations.

We have now calculated and added the correlation between measured and estimated zinc deficiency country data:

Estimated zinc deficiency prevalences were derived from dietary zinc intake using national food balance sheets, making them more reflective of nutritional factors [1,39,40]. Note that, based on the 13 complete observations, measured and estimated zinc deficiency prevalences were positively correlated (Pearson correlation = 0.46; one-sided p-value = 0.05).

7) The authors explore one hypothesis for positive selection on SLC30A2 but fail to mention other, more prevalent hypotheses for genetic adaptation in zinc transporters (see literature mentioned in point 3). They should more thoroughly discuss potential mechanisms for driving allele frequency differences amongst populations (e.g., ancestral zinc levels in soils, ancestral diet, pathogen-starvation ..).

The reviewer is correct and we toned down every mention of positive selection in the manuscript:

Considering that the SLC30A2/ZnT2 transporter is responsible for zinc secretion in human milk, we hypothesized that this correlation may be induced by a possible selection mechanism for offspring survival in regions with elevated prevalence of zinc deficiency [...]

and hinting at possible evolutionary signatures of natural selection to maintain the equilibrium of zinc homeostasis

We have also added various other causes that can lead to the observed geographic correlations (see above and also part of the limitations):

Furthermore, other potential mechanisms can also give rise to such geographic correlations amongst populations, including different ancestral zinc levels in soils, distinct ancestral diet, divergent socio-economic conditions or differences in pathogen-induced zinc starvation. More in-depth analysis separating these effects necessitates future research, as was conducted for selenium [https://doi.org/10.1093/molbev/msy070], but it is out of the scope of our current investigation.

8) The authors should also more explicitly recognise that a) polygenic traits often have more complex architecture in sub-Saharan African populations and b) there is poor portability between GWAS studies conducted on individuals of European-ancestry and individuals of diverse sub-Saharan-African diversity. The GRS calculated is therefore likely a poor approximation of urinary zinc levels in the global areas of highest zinc deficiency.

Indeed, the PRS based on European samples is a sub-optimal proxy for urinary zinc levels in non-European populations. Since we do not have access to a sufficient number of genotyped non-European samples with urinary zinc data available, we cannot test this. Nevertheless, PRSs for any explored trait has on average >25% explained variance compared to European samples [https://doi.org/10.1038/s41588-019-0379-x], thus there is still a non-negligible predictive power. But we fully acknowledge this limitation in the updated discussion:

First, we used data from European cohorts, where the prevalence of dietary zinc deficiency is generally low [1], which precludes determining the exact health-effects resulting from an increased urinary zinc excretion and its associated genetic variants. As a consequence, the ecological analysis is error-prone as it assumes that the effect of the lead variants is similar in European and other populations.

Reviewer #2: The authors present the first GWAS focused on urinary zinc levels, filling a gap in current zinc homeostasis research. This is a highly promising and well-executed study and the integration of genetics, epidemiology, and experimental models is commendable. I have no major concerns with this manuscript.
Please find some minor comments below

We thank the reviewer for the positive assessment of our work and the comments aimed at ensuring the robustness of the Mendelian randomization and finemapping results. Please find below the answers to their comments.

- Consider add F-statistics to Table 4.

We thank the reviewer for the suggestion. We calculated the F-statistics for the forward and reverse Mendelian randomization analyses, by using the Cragg-Donald formula (Burgess et al., 2011; doi: 10.1093/ije/dyr036). For both the forward and reverse results, we obtained high F-statistics (>10) suggesting that the level of weak instrument bias is low. We updated the result table accordingly.

Table 4: Bidirectional Mendelian randomization (MR) results between urine zinc concentrations and quantitative/disease phenotypes. Forward MR results quantify the causal effect of urinary zinc concentrations on the trait (b_{MR}-forward), and reverse MR results the causal effect of the trait on urinary zinc concentrations (b_{MR}-reverse). b_{MR} - MR causal effect estimated through the inverse-variance weighting method (SD/SD); n_{IV} - number of instrumental variables; Q_{HET} p-value - p-value from a Cochran's Q-statistic heterogeneity test; F_{stat} - F-statistics quantifying the strength of association between the instrumental variables and the exposure; T2D - type 2 diabetes; CAD - coronary artery disease; SBP - systolic blood pressure; DBP - diastolic blood pressure; LDL-C - low-density lipoprotein cholesterol; TC - total cholesterol; TG - triglycerides; CRP - C-reactive protein.

Trait	b_{MR} (forward)	p-value$_{MR}$ (forward)	n_{IV} (forward)	Q_{HET} p-value (forward)	F_{stat} (forward)	b_{MR} (reverse)	p-value$_{MR}$ (reverse)	n_{IV} (reverse)	Q_{HET} p-value (reverse)	F_{stat} (reverse)
T2D	0.001	9.40E-01	11	2.09E-15	102.3	0.135	3.38E-04	315	2.00E-04	67.8
HbA1c	0.029	2.94E-01	11	2.22E-59	102.3	-0.023	3.79E-01	733	2.28E-09	91.9
Glucose	0.015	3.72E-01	11	9.98E-17	102.3	0.056	2.75E-01	168	1.73E-02	89.2
CAD	0.020	1.25E-01	9	3.53E-09	116.9	0.027	6.13E-01	226	1.25E-01	65.8
SBP	0.016	1.71E-01	11	3.32E-07	102.3	0.078	9.19E-02	374	3.51E-02	48.3
DBP	-0.001	9.76E-01	11	1.89E-23	102.3	0.135	1.36E-02	319	1.38E-06	50.1
TC	0.047	1.22E-01	11	1.31E-69	102.3	0.006	8.46E-01	391	1.91E-05	114.3
LDL-C	0.045	1.22E-01	11	1.65E-62	102.3	-0.017	5.82E-01	368	1.82E-04	121.5
TG	-0.034	1.59E-02	11	3.25E-12	102.3	0.139	7.91E-08	591	4.02E-03	102.0
Stroke	0.031	2.15E-03	9	1.17E-01	116.7	-0.378	3.56E-02	11	6.96E-01	41.1
CRP	0.027	9.90E-02	11	2.77E-18	102.3	-0.053	6.06E-02	488	1.45E-02	100.0
Height	-0.021	2.76E-01	11	1.96E-27	102.3	0.013	3.89E-01	2294	1.91E-02	75.7
Bone mineral density	-0.018	2.38E-01	11	3.49E-08	102.3	0.037	9.70E-02	581	2.94E-01	75.5
Calcium	0.012	6.46E-01	11	3.58E-46	102.3	0.085	9.93E-03	415	1.44E-01	76.0
Birthweight	0.000	9.70E-01	11	4.67E-04	102.3	-0.027	6.80E-01	126	6.22E-03	50.1

- It's not clear why MR egger results are not included.

We have updated the bidirectional MR sensitivity analyses to not only include MR median and mode, but also MR Egger. The MR relationships that were identified to be significant in the inverse-variance weighting methods, also remained significant in the MR Egger analyses. The numerical results were added to Table S3 and S5, and the result section was updated as follows:

To examine whether the observed phenotypic associations could mean causal relationships, we performed bidirectional Mendelian randomization analyses. These revealed a (Bonferroni) significant (<0.05/15 = 3.3E-03), but modest, forward causal effect of increased urinary zinc levels on increased stroke risk (b_{MR} = 0.031; p-value = 2.15E-03; Table 4) suggesting that the observed epidemiological association may be driven by a true causal link. Given the high pleiotropy [...]. Although the causal effect with stroke did not reach significance when applying the simple mode and median MR methods, likely due to the low number of IVs, the causal effect on stroke remained robust in the MR Egger analysis (p-value = 0.01) and after removing ABO and SCL39A8 associated IVs (Table S4).

Inspecting reverse causal relationships, we found that increased genetic liability to type 2 diabetes and elevated triglyceride levels, increased zinc excretion (b_{MR} = 0.135, p-value = 3.38E-04 and b_{MR} = 0.139, p-value = 7.91E-08, respectively), which remained significant in the Egger, simple median and mode MR methods (Table S5). [...].

- Suggest to also add IVW regression lines to supp figure 13-15.

We have updated Supplementary Figures 13-15 to include MR-IVW regression lines.

- The authors note strong heterogeneity in effect sizes for rs3008217 GWAS associations across cohorts. It may be worth performing fine mapping prior to meta-analysis for this locus.

We thank the reviewer for this suggestion and have now performed fine-mapping in each cohort separately to assess whether distinct causal SNPs are identified. The cohort-specific fine-mapping results have been added to a new Dataset S3 file, and we have updated the results section "*Genetic loci associated with urinary zinc excretion*" accordingly to include the new results:

Three credible sets (CS) were identified and within these sets we identified the SNPs with the highest probability of being causal. This was assessed by estimating the highest posterior inclusion probability (PIP), a value between zero and one, with zero representing no support and one corresponding to 100% certainty. At this locus the following three SNPs were the most likely causal variants: rs11587475 (PIP = 0.38, CS = 1, 3'UTR variant of SLC30A2), rs3008217 (PIP = 0.27, CS = 2, intronic variant of a lncRNA ENSG00000284309) and rs17257086 (PIP = 0.09, CS = 3, intronic variant of TRIM63).

Given the heterogeneity of effect sizes at this locus, we performed fine-mapping in each cohort separately. While rs3008217 was the top SNP in the meta-analysis and CoLaus|PsyCoLaus, GCKD and SKIPOGH identified different lead SNPs with the highest PIP (rs6686190 with PIP = 0.14 in GCKD and rs3008208 with PIP = 0.25 in SKIPOGH; Dataset S3). Together with the modest PIP of 0.38 in the meta-analysis, this indicates low confidence in rs3008217 being the causal variant and suggests the presence of an alternative, possibly unmeasured, causal SNP driving the SLC30A2 signal.
